# Membrane stiffness is modified by integral membrane proteins†
†Electronic supplementary information (ESI) available. See DOI: 10.1039/c6sm01186a



**DOI:** 10.1039/c6sm01186a

**Published:** 2016-08-26

**Authors:** Philip W. Fowler, Jean Hélie, Anna Duncan, Matthieu Chavent, Heidi Koldsø, Mark S. P. Sansom

**Affiliations:** a Department of Biochemistry , University of Oxford , South Parks Rd , Oxford , OX1 3QU , UK . Email: mark.sansom@bioch.ox.ac.uk ; Fax: +44 01865 613238 ; Tel: +44 01865 613306

## Abstract

The ease with which a cell membrane can bend and deform is important for a wide range of biological functions. Peripheral proteins that induce curvature in membranes (*e.g.* BAR domains) have been studied for a number of years. Little is known, however, about the effect of integral membrane proteins on the stiffness of a membrane (characterised by the bending rigidity, *K*_c_). We demonstrate by computer simulation that adding integral membrane proteins at physiological densities alters the stiffness of the membrane. First we establish that the coarse-grained MARTINI forcefield is able to accurately reproduce the bending rigidity of a small patch of 1500 phosphatidyl choline lipids by comparing the calculated value to both experiment and an atomistic simulation of the same system. This enables us to simulate the dynamics of large (*ca.* 50 000 lipids) patches of membrane using the MARTINI coarse-grained description. We find that altering the lipid composition changes the bending rigidity. Adding integral membrane proteins to lipid bilayers also changes the bending rigidity, whilst adding a simple peripheral membrane protein has no effect. Our results suggest that integral membrane proteins can have different effects, and in the case of the bacterial outer membrane protein, BtuB, the greater the density of protein, the larger the reduction in stiffness.

## Abstract 2

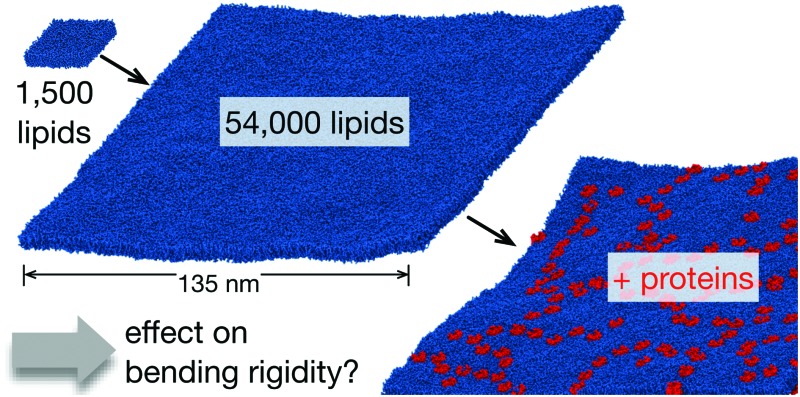
Large coarse-grained simulations show that integral membrane proteins alter the bending rigidity of lipid bilayers.

## 1 Introduction

Cell membranes are complex structures formed by bilayers containing many different species of lipids, into which are inserted a wide range of proteins, frequently at high concentrations.^1,2^ Their main function is to provide a selective molecular barrier, thereby enabling the formation of distinct compartments, including cells and their organelles. The dynamic and elastic properties of membranes are important for a wide range of biological functions including endo- and exocytosis, cell division, autophagy, tubulation, and the clustering of cell signalling proteins to enhance signal transmission.^3^

The ease with which a membrane can be perturbed governs not only the magnitude of its thermal fluctuations at equilibrium, but also the ease with which the membrane can be deformed or sculpted by the action of proteins. Several classes of proteins that induce large changes in the local curvature of membranes, for example BAR domains, have been identified and extensively studied, both experimentally^4–6^ and computationally.^7,8^ Some proteins that less strongly affect the local organisation of the membrane, for example, the yeast or human vesicle trafficking protein Sar1 (which has an amphipathic N-terminus which inserts into the membrane surface) have nonetheless been shown to reduce the macroscopic stiffness of the membrane.^9,10^ In addition, there are a number of experiments showing that certain peptides and long polymers reduce the stiffness of lipid bilayers.^11–15^

Rather less attention has been focussed on the effect of the presence of non-sculpting integral membrane proteins on the macroscopic stiffness of cell membranes: one study of bacteriorhodopsin showed that it had no effect on the stiffness of the membrane,^16^ whilst another study demonstrated that the presence of a Ca^2+^ATP-ase pump, SERCA1A, in giant unilamellar vesicles decreased the stiffness of the membrane, *K*_c_.^17^ The latter effect was ascribed to the conical shape of the protein. Interestingly, it has also been demonstrated that activating both proteins also decreases the stiffness of the membrane.^17,18^ Investigating whether the presence of integral proteins in a membrane at physiological concentrations affects the stiffness of lipid bilayers is the key aim of this paper. We note that there is a converse problem: investigating how the propensity of lipids to form different curved surfaces affects the function of individual membrane proteins.^19^ The latter, however, is not the focus of this paper.

To measure how easily a membrane can be distorted a theory with measurable parameters is needed. The simplest candidate is provided by Helfrich–Canham (HC) elastic theory,^20–22^ which models a membrane as a continuous elastic sheet. Assuming planarity, the fundamental parameter in this theory that describes the stiffness of the membrane is the bending rigidity (*K*_c_). A wide range of experimental techniques have been developed^23,24^ which use this theory to estimate values of *K*_c_.^25,26^ It has proved challenging to measure *K*_c_ experimentally^26^ and there can be up to an order of magnitude of difference between values of *K*_c_ estimated using different techniques,^27^ reflecting the difficulty of measuring the elasticity of membranes. Several enhancements to HC theory have been proposed, but, as yet, there is no clear candidate that is able to explain and model all aspects of the observed behaviour, and therefore replace HC theory in the interpretation of experiments. The theoretical literature on the effect of adding proteins, often referred to as inclusions, is unclear with some studies predicting an increase in the stiffness of the lipid bilayer,^28^ whilst others predict a decrease.^29,30^ As there is currently no theoretical consensus and since all reported values of bending moduli assume the validity of Helfrich–Canham theory, we shall therefore use simple HC theory to interpret our simulations.

**Fig. 1:**
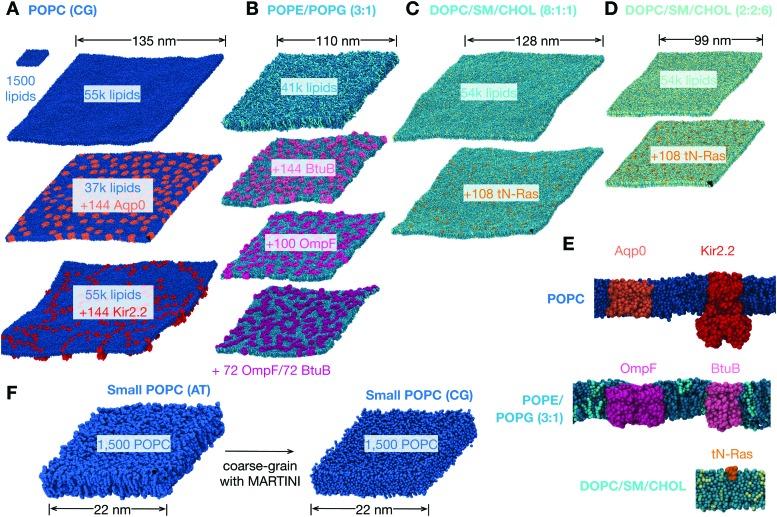
The lipid bilayers and membrane proteins studied using the MARTINI2.2 coarse-grained forcefield^42,43^. (A) Three large POPC bilayers were simulated; one of 54 684 lipids without protein, a slightly smaller bilayer with 37 249 lipids and 144 copies (29% by area) of an aquaporin, Aqp0^44^, and a slightly larger bilayer with 55 584 lipids and 144 copies (11%) of the inward-rectifying potassium channel, Kir2.2^45^. (B) A simple two component lipid bilayer comprising POPE/POPG in the ratio 3 : 1 was also simulated. To test the effect of protein either 144 copies (28% by area) of the vitamin B_12_ transporter BtuB, 100 copies (37%) of the outer membrane protein F, OmpF, or 72 of each protein (40%) were inserted as shown. Finally a slightly more complex ternary mixture of lipids (DOPC, sphingomyelin (SM) and cholesterol) was studied. Two different compositions of DOPC : SM : cholesterol were analysed. (C) A low cholesterol mixture in the ratio 8 : 1 : 1 and (D) a high cholesterol mixture in the ratio 2 : 2 : 6. The effect of protein on each was assessed by adding 108 copies (<1% by area) of the truncated cell signalling protein, tN-Ras. (E) Images of a single copy of each of the five proteins considered in this study. For additional images see Fig. S1 (ESI†). (F) To validate our use of the MARTINI coarse-grained (CG) forcefield, a patch of 1500 POPC lipids was simulated for 0.5 μs using the CHARMM36 atomistic (AT) forcefield and for up to 5 μs using the MARTINI2.2 forcefield.

Computer simulation, mainly molecular dynamics (MD), has been successfully used to study the elastic properties of lipid bilayers.^31–35^ More recently, the advent of coarse-grained descriptions of lipids have allowed significantly larger patches of lipids to be simulated.^36–39^ In this paper we use computer simulation to demonstrate how either altering the lipid composition or including membrane proteins at concentrations comparable to those present in cell membranes *in vivo* affects the bending rigidity of a series of simple membrane models. These range from single lipid component bilayers to more complex ternary mixtures of lipids (Fig. 1A). Finally we shall extend our analysis to a complex membrane model, comprising multiple single transmembrane helices embedded within a bilayer whose lipid composition mimics that of a mammalian plasma membrane.^40,41^

## 2 Results

We chose lipid mixtures that are very simple models of an eukaryotic membrane (pure POPC), an *E. coli* membrane (3 : 1 POPE/POPG) and a ternary mixture (DOPC/sphingomyelin/cholesterol) that is used *in vitro* to study disordered and ordered phases of membranes.^46^ One or two proteins appropriate for each of these bilayers were chosen (Fig. 1B and Fig. S1, ESI†). For the simple model of an eukaryotic membrane, we studied the effect of inserting either an aquaporin, Aqp0, that forms water-conducting pores or an inwardly-rectifying potassium channel, Kir2.2, that is involved in regulating the resting electrical potential of cells. The effect of inserting either (or both) of two different outer membrane proteins, the vitamin B_12_ transporter BtuB and the porin OmpF, into the simple binary model of an *E. coli* membrane was also examined. Finally, we determined the effect of adding the truncated form of a human small G-protein, N-Ras, to two different mixtures of DOPC, sphingomyelin and cholesterol.

### 2.1 Theoretical background

**Fig. 2:**
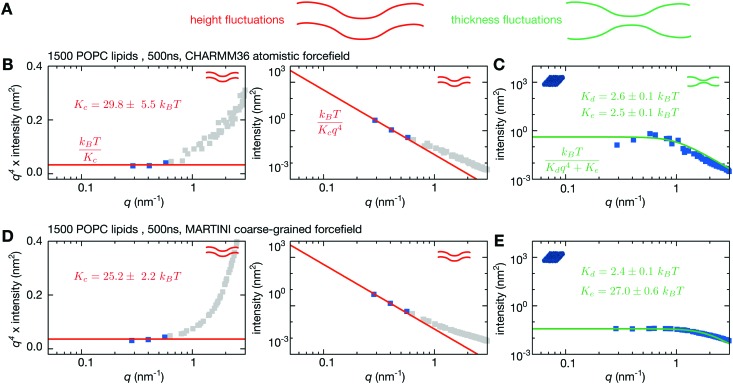
A coarse-grained simulation of 1500 POPC lipids yields similar height fluctuations to a simulation run using an all-atom forcefield. (A) Both the height and thickness of a lipid bilayer can fluctuate. (B) The power spectra of the height fluctuations for the all-atom simulation of the bilayer are plotted as grey squares on a log–log plot. The value of *K*_c_ is fitted on a plot of *q* ^4^ × intensity of the height spectrum *vs*. *q*. This is more accurate since all points fitted have equal weight. Since Helfrich–Canham (HC) theory is only valid for small *q*, only values with *q* < 0.6 nm^–1^ are considered – these are coloured blue. The fit is drawn as a red line and the value of *K*_c_ is predicted to be 29.8 ± 5.5*k*_B_ *T*. This is then plotted on the conventional power spectrum of the height fluctuations. (C) The HC form for the thickness fluctuations is fitted directly onto the power spectrum of the thickness fluctuations (shown by the green line) yielding *K*_d_ = 2.6 ± 0.1*k*_B_ *T* and *K*_e_ = 2.5 ± 0.1*k*_B_ *T*. The same analysis is repeated for a MARTINI coarse-grained simulation of the same duration. (D) *K*_c_ is predicted to be 25.3 ± 1.8*k*_B_ *T* and therefore agrees within error. (E) By contrast, the power spectrum of the thickness fluctuations is different, which is reflected in values of *K*_d_ = 2.4 ± 0.1*k*_B_ *T* (which agrees with the atomistic simulation) and *K*_e_ = 26.7 ± 0.7*k*_B_ *T* (which is ∼10× larger than the atomistic simulation). Convergence times and errors are calculated as described in the Methods and the (Fig. S2 and S3, ESI†).

Helfrich–Canham (HC) theory relates the equilibrium fluctuations of a membrane to its elastic properties. Two main fluctuation modes are possible; variation in the height of the membrane surface above some reference plane and variation in the thickness of the bilayer (Fig. 2A). In the absence of surface tension, the power spectrum of the height fluctuations (also called undulatory motions), 〈|*h*(**q**)|^2^〉, is predicted to be given by1
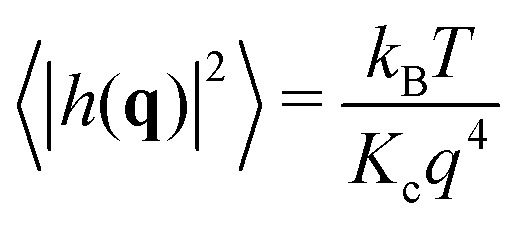
where *q* is the magnitude of the wavevector **q** (*i.e.* the wavenumber, nm^–1^), *K*_c_ is the bending rigidity, *T* is the temperature and *k*_B_ is Boltzmann's constant.^20,21^ Since HC theory models the bilayer as an elastic sheet, this expression is only valid at lengthscales much longer than the thickness of the bilayer (*i.e.* small wavenumbers, *q*). Calculating the power spectrum of the height fluctuations of a lipid bilayer and fitting the above equation is the obvious route to estimating the value of *K*_c_ for a lipid bilayer. The resulting fit, however, ends up being dominated by the first few points which have very large intensities. Instead it is more accurate to rearrange and fit to this expression2
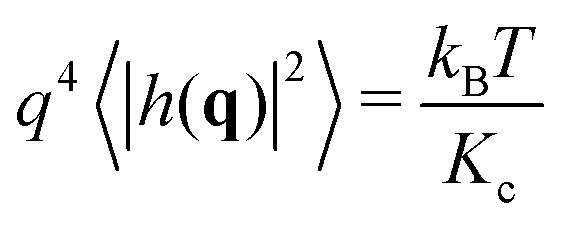
where each point contributes equally. We shall therefore plot *q* ^4^〈|*h*(**q**)|^2^〉 against *q* for each simulation and fit the constant, *k*_B_ *T*/*K*_c_, in the low-*q* region. For small, medium and large membrane patches this is taken to be *q* < 0.6, 0.4 and 0.2 nm^–1^, respectively.

Helfrich–Canham theory predicts that the power spectrum of the thickness fluctuations (also called peristaltic motions) is given by3
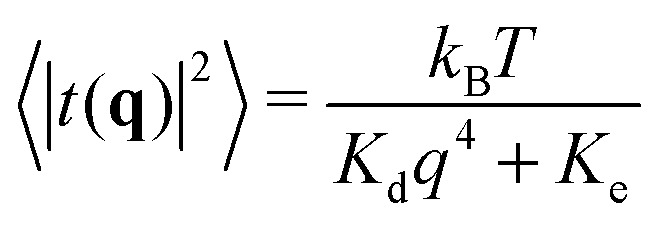
where *K*_d_ is the elastic rigidity of peristaltic modes and *K*_e_ is a harmonic force constant describing the ease with which the membrane thickness can be perturbed.^33^ In the limit of low *q* this simplifies to4
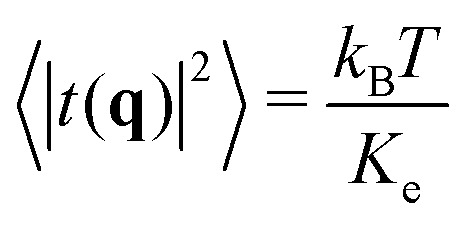
 which is a constant, and therefore we expect the power spectrum of the thickness fluctuations to approach an asymptote at low *q*.

We note that there are several enhancements to HC theory that may better describe our power spectra. The first takes into account how easy it is for individual lipids to tilt away from the local membrane normal by introducing a second elastic constant.^47–49^ This tilt-dependent theory has been shown to better describe the power spectrum of height fluctuations at both low and high values of *q* for simple pure lipid bilayers^48–50^ and there is some early experimental justification for this approach.^51^ As we shall see shortly, when we add proteins to our bilayers, the power spectra become more complex and hence, although this theory may better describe our results for pure lipid bilayers, it cannot describe the majority of power spectra we observe for bilayers containing proteins and therefore we shall not apply it here.

The second enhancement takes into account the coupling between undulatory and peristaltic modes, leading to more complex expressions for the relevant power spectra.^52^ These expressions require a minimum of five parameters, as opposed to HC theory which only requires three. It is possible that this theory may be able to describe the maxima in the power spectra of the thickness fluctuations that we occasionally see. However, since that is not the focus on this paper, we shall proceed with the accepted Helfrich–Canham theory as given by eqn (1) and (4) for the sake of simplicity.

### 2.2 Atomistic simulation accurately models the bending rigidity of a POPC lipid bilayer

Using molecular dynamics we simulated a patch of 1500 POPC lipids using the fully-atomistic CHARMM36 forcefield^53^ for 0.5 μs (Fig. 1F, Table 1 and Table S1, ESI†). This is larger than nearly all previous studies using atomistic simulations of a lipid bilayer – a simulation of 1600 sphingomyelin lipids was run over ten years ago, but only for 4 ns.^34^ A previous study has demonstrated that CHARMM36 can accurately simulate the behaviour of lipids in different phases.^35^ The resulting power spectra of the height and thickness fluctuations are shown in Fig. 2B and C. As expected, the undulatory modes have higher intensities at lower values of *q* whereas the intensity of the thickness modes appear to be converging to a plateau at low *q*.

**Table 1:** The bending rigidity, *K*_c_, for each of the simulations. Errors were calculated, including convergence testing, as described in the ESI (Fig. S2–S5). All the coarse-grained simulations were run at 323 K, with the exception of the two component POPE/POPG bilayers which were run at 313 K. The protein area density was estimated by calculating the average area of the last 20% of each simulation and inferring the protein area using the appropriate area per lipid value calculated from the control simulations

Sim no.	Lipid composition	No. lipids	Forcefield	Duration (μs)	Protein	Area density	*K*_c_ (kT)
1	POPC	1500	AT	0.5	—	—	29.8 ± 5.5
2		1500	CG	0.5	—	—	25.2 ± 2.2
				5	—	—	25.5 ± 0.5
3		54 684	CG	5	—	—	30.9 ± 1.3
4		37 249	CG	5	+144 Aqp0	29%	37.3 ± 3.6
5		55 584	CG	5	+144 Kir2.2	11%	16.1 ± 0.7

6	POPE/POPG 3 : 1	41 472	CG	5	—	—	21.2 ± 1.8
7		28 888	CG	5	+144 BtuB	28%	10.2 ± 1.1
8		26 832	CG	5	+100 OmpF	37%	19.4 ± 2.2
9		25 448	CG	5	+72 BtuB/72 OmpF	40%	16.6 ± 1.2

10	DOPC/SM/CHOL 8 : 1 : 1	53 964	CG	5	—	—	25.0 ± 2.0
11		53 964	CG	5	+108 tN-Ras	<1%	23.8 ± 2.2

12	DOPC/SM/CHOL 2 : 2 : 6	53 964	CG	5	—	—	49.1 ± 6.7
13		53 964	CG	5	+108 tN-Ras	<1%	51.1 ± 2.9

Fitting eqn (2) to a graph of *q* ^4^〈|*h*(**q**)|^2^〉 against *q* (Fig. 2B) yields a value of *K*_c_ = 29.8 ± 5.5*k*_B_ *T*. This compares favourably with experiment; two different aggregated datasets of experimentally-determined values of *K*_c_ for pure POPC bilayers span the range 5.8–49*k*_B_ *T* with averages of 19 and 27*k*_B_ *T*, respectively.^23,27^ The power spectrum of the height fluctuations at high values of *q*, however, is not well described by HC theory (eqn (1)), confirming that the theory is not valid at high *q*. The power spectrum of the thickness fluctuations is moderately well described by eqn (4) but shows evidence of a maximum around *q* ∼ 0.6 nm^–1^ (Fig. 2C). From fitting eqn (4) we estimate *K*_d_ and *K*_e_ to be 2.6 ± 0.1*k*_B_ *T* and 2.5 ± 0.1*k*_B_ *T*, respectively. These values are difficult to validate since there are few experimental data available.

This simulation is relatively small and therefore only samples fluctuations down to *q* ∼ 0.3 nm^–1^. Hence there are only a few datapoints where eqn (1) holds, leading to fitting difficulties and a relatively large error in the value of *K*_c_. If we are to better characterise the dynamic behaviour of lipid bilayers, we need to sample their behaviour at lower values of *q*, which requires simulations of much larger patches of lipid bilayers. Since it is extremely challenging at present to run atomistic (AT) simulations of bilayers containing the tens of thousands of lipids required to probe down below *q* ∼ 0.1 nm^–1^ we shall therefore switch to a coarse-grained (CG) description of the lipids and waters.

### 2.3 Coarse-grained simulations reproduce the elastic properties of atomistic simulations

Coarse-graining has proved a successful method to model the behaviour of soft condensed matter systems,^54,55^ such as lipid bilayers,^40,41^ membrane vesiculation^56^ and virus dynamics^57,58^ and budding.^59^ In this work we shall use the MARTINI2.2 forcefield:^42,43^ since this replaces only every four heavy atoms by a single bead, it retains more chemical accuracy than some schemes which are even more coarse-grained. Before we can go on to examine the behaviour of large coarse-grained lipid bilayers it is first necessary to establish that lipid bilayers modelled using the MARTINI forcefield accurately reproduce the long-wavelength dynamics that underlie the stiffness of a membrane. We therefore simulated an identical patch of 1500 POPC lipids, but instead using MARTINI2.2, for 500 ns.

The power spectrum of the height fluctuations of the coarse-grained POPC bilayer is very similar to that of the atomistic bilayer (Fig. 2D), except at larger values of *q*. Such a difference at larger values of *q* is perhaps to be anticipated given the nature of the coarse-graining approach. Fitting the form predicted by HC theory (eqn (2)) yields a value for *K*_c_ of 25.2 ± 2.2*k*_B_ *T*, in agreement with both our previous simulation and published experimental values.^23,27^ The power spectrum of the thickness fluctuations, however, is notably different, asymptoting to a significantly lower intensity as *q* → 0. This difference is mainly reflected in a ∼10× larger value of *K*_e_ which is a measure of how easy it is separate the two leaflets of the bilayer, indicating that the leaflets of a bilayer are held more tightly together in MARTINI2.2 than CHARMM36. We conclude that the MARTINI2.2 forcefield is sufficiently accurate to model the long-wavelength undulatory dynamics of a lipid bilayer, as described by the bending rigidity (*K*_c_). This is not surprising since MARTINI was originally parameterised to reproduce the bending rigidity for a related PC lipid,^60^ however it is reassuring that this behaviour is maintained at significantly larger lengthscales.

**Fig. 3:**
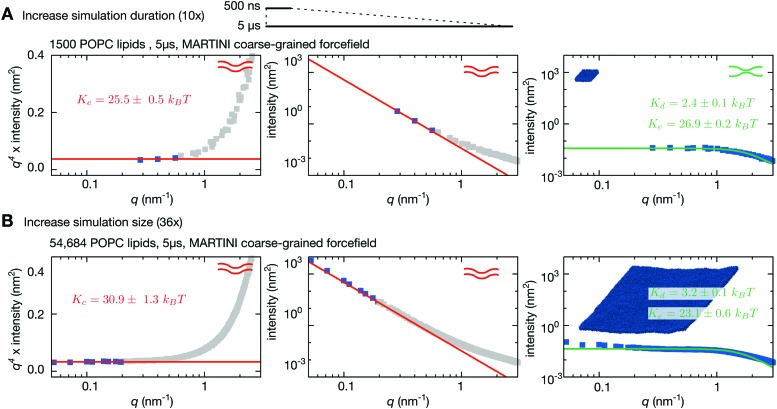
Increasing the size of the lipid bilayer increases the precision of the predictions. (A) Increasing the simulation duration by 10× to 5 μs does not significantly alter the predicted values of *K*_c_, *K*_d_ & *K*_e_. (B) Increasing the size of the bilayer patch by a factor of 36 allows longer wavelength (lower-*q*) fluctuations to be sampled resulting in agreement with Helfrich–Canham theory over a wider range of *q* values. Due to the larger size, the fits are carried out for *q* < 0.2 nm^–1^ and result in a value of *K*_c_ = 30.9 ± 1.3*k*_B_ *T*. Convergence times and errors are calculated as described in the Methods and the (Fig. S2 and S3, ESI†).

One major concern when studying long lengthscale dynamics is whether they have converged. To test this we extended the simulation of the coarse-grained patch of 1500 POPC lipids by a factor of ten to 5 μs. Repeating the same analysis, confirmed that the original simulation was converged (Fig. S2 and S3, ESI†) and yielded values of *K*_c_, *K*_d_ & *K*_e_ that were within the error of the previous values (Fig. 3A and Table 1). Next we studied the effect of increasing the size of the membrane patch by simulating a POPC lipid bilayer over 36 times larger in surface area, having 54 684 lipids in total (Fig. 1A, Table 1 and Table S1, ESI†). This is nearly seven times larger than the previous largest MARTINI simulation of a lipid bilayer used to study fluctuations.^61^ Comparing the power spectra to those calculated from the smaller patch of POPC lipids (Fig. 3) shows that, as anticipated, the larger bilayer has identical fluctuations to the smaller bilayer at intermediate and high values of *q* but, in addition, samples down to *q* ∼ 0.05 nm^–1^. Sampling the intensities of the height power spectrum over a wider range of values of *q* allows us to better test how well HC theory describes the behaviour observed in the simulations. Eqn (1) describes well the power spectrum of the height fluctuations for about an order of magnitude of *q*, predicting that *K*_c_ = 30.9 ± 1.3*k*_B_ *T* (Fig. 3B and Table 1), in good agreement with our previous estimates and experimental data.^23,27^ Interestingly, the intensities in the power spectrum of the thickness fluctuations start increasing again below *q* = 0.1 nm^–1^, which disagrees with the behaviour predicted by HC theory (eqn (4)).

### 2.4 Lipid composition modulates bilayer stiffness

**Fig. 4:**
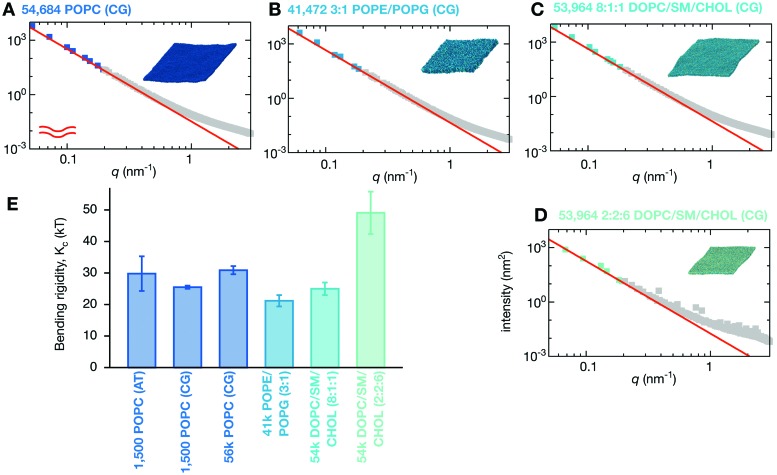
Changing the lipid composition alters the bending rigidity of the bilayer. (A) For comparison the power spectrum of the height fluctuations of the large POPC bilayer is shown. This and all other fits are derived from Fig. S6 (ESI†) which weights each point equally. The analysis is repeated for (B) the two-component POPE/POPG (3 : 1) bilayer and the three-component DOPC/sphingomyelin/cholesterol bilayers in (C) low-(8 : 1 : 1) and (D) high-cholesterol (2 : 2 :6) mixtures. (E) A bar chart showing the variation in calculated values of the bending rigidity, *K*_c_. Error bars are drawn. Convergence times and errors are calculated as described in the Methods and the (Fig. S4 and S5, ESI†). The power spectra of the thickness fluctuations can be found in Fig. S7 (ESI†).

What happens when we simulate a lipid bilayer with more than one lipid species? First let us consider a simple two-component symmetric lipid bilayer with 75% POPE and 25% of the anionic lipid POPG – this is a simple model of the lipid composition of a bacterial (*E. coli*) membrane. Although one might expect the net charge on the bilayer to damp down the fluctuations,^62^ the power spectrum of the height fluctuations is similar to the pure POPC lipid bilayer, and is therefore well described by HC theory, with *K*_c_ calculated to be 21.2 ± 1.8*k*_B_ *T*, a reduction of 31% compared to the pure POPC lipid bilayers (Fig. 4B and Table 1). Unlike POPE and POPC which have a positively-charged ‘headgroup’ bead and a negatively-charged ‘phosphate’ bead, POPG has a neutral headgroup bead and a negatively-charged ‘phosphate’ bead, and therefore a quarter of the headgroup beads in the POPE/POPG bilayer will be uncharged. The reduction in the bending rigidity is hence possibly due to a decrease in the electrostatic repulsion between the neighbouring headgroup beads when they form a concave surface, allowing the membrane to flex more easily. The long-wavelength fluctuations in the heights of these lipid bilayers occur on approximately the sub-microsecond timescale (see the ESI,† Movies).

Although it is generally accepted that cholesterol increases the stiffness of membranes, this is not true for DOPC up to a concentration of 40%.^63,64^ We therefore simulated a ternary mixture of DOPC, sphingomyelin and cholesterol and examined the effect of increasing the cholesterol concentration. This ternary mixture is often used experimentally in giant unilamellar vesicles to create disordered and ordered lipid bilayers by altering the relative lipid concentrations.^46^ We first made a ternary mixture that was a minor departure from our previously studied lipid bilayers with only 10% cholesterol, 10% sphingomyelin and 80% DOPC. Again the power spectrum of the height fluctuations was very similar to the control POPC lipid bilayer (Fig. 4C), resulting in a value of *K*_c_ of 25.0 ± 2.0*k*_B_ *T* (Table 1), a reduction of 19%. The bending rigidity, *K*_c_, of a similar mixture with 20% cholesterol, 10% sphingomyelin and 70% DOPC was measured experimentally to be 23.7 ± 1.2*k*_B_ *T*,^65^ *i.e.* within error. This mixture is therefore slightly easier to deform than the control POPC lipid bilayer.

Increasing the concentration of cholesterol to 60% (with 20% each of DOPC and sphingomyelin) yields a more ordered lipid bilayer, which we can infer from the reduction of the lateral dimension of the bilayer from ∼130 nm to ∼100 nm (Fig. 1A and Table S1, ESI†). This is an unphysiologically high concentration of cholesterol, although this composition is used *in vitro* to create ordered phases.^46^ The bending rigidity is predicted to be 49.1 ± 6.7*k*_B_ *T* (Fig. 4D), roughly double that of the low cholesterol mixture. The increase in stiffness, and also the reduction in the rates at which both proteins and lipids move, can be seen visually (see ESI,† Movies).

### 2.5 Inclusion of transmembrane proteins alters the stiffness of membranes

**Fig. 5:**
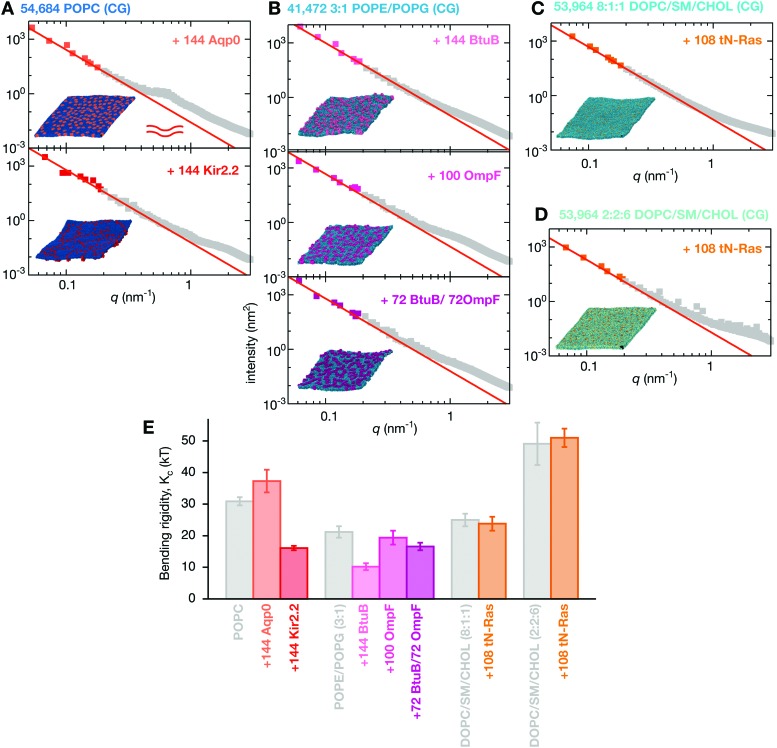
Integral membrane proteins tend to increase the magnitude of fluctuations of the bilayer. (A) Inserting 144 copies of Aqp0, an aquaporin, or Kir2.2, an inward-rectifying potassium ion channel, produces a membrane that obeys Helfrich–Canham (HC) theory at low *q*, however, the Aqp0 proteins lead to a pronounced hump in the intensity around *q* ∼ 4 nm^–1^. These and all other fits are derived from Fig. S6 (ESI†). (B) POPE/POPG (3 : 1) bilayers which have had the bacterial proteins BtuB or OmpF or both proteins inserted are also well described by HC theory at low *q*. Likewise, adding 108 copies of the truncated peripheral cell signalling protein tN-Ras to either the (C) low or (D) high cholesterol ternary lipid mixtures produces a membrane whose dynamics are well described by HC theory. (E) The calculated values of the bending rigidity, *K*_c_, show the integral membrane proteins (Kir2.2, BtuB & OmpF) all reduce the stiffness of the bilayers, the integral membrane protein Aqp0 increases the stiffness slightly (2*σ*) whereas the peripheral membrane protein, tN-Ras, has no effect on the stiffness of the ternary lipid mixture. Convergence times and errors are calculated as described in the Methods and the (Fig. S4 and S5, ESI†). The power spectra of the thickness fluctuations can be found in Fig. S7 (ESI†).

Next we examined the effect of including integral membrane proteins in these lipid bilayers. We inserted 144 copies of either an aquaporin, Aqp0, or an inwardly-rectifying potassium ion channel, Kir2.2, into the pure POPC lipid bilayer. These mammalian proteins occupied 29% and 11%, respectively of the bilayer surface area: for comparison proteins occupy at least 23% of the area of the membrane of red blood cells^1^ and 20% of the area of membranes of synaptic vesicles.^2^ Adding Aqp0 introduces a shoulder to the power spectrum of the height fluctuations at *q* ∼ 0.7 nm^–1^ (Fig. 5A). This cannot be explained by the newer tilt-dependent theories discussed earlier,^47–49^ hence we fit Helfrich–Canham theory (eqn (1)) at low *q* (Fig. S6, ESI†), leading to *K*_c_ = 37.3 ± 3.6*k*_B_ *T*, which is an increase in stiffness of 21%, however this is only just significant at 2*σ*. In addition, the fit of eqn (2) at low-*q* (Fig. S6, ESI†) is markedly less good than the pure lipid bilayers, suggesting that the simulation may not yet be converged (for example the proteins may be beginning to cluster), larger patch sizes may be required to reach the *q* ^4^ regime or a new theory may be required to deal with the presence of protein at physiological concentrations.

The undulatory modes of the Kir2.2 simulation appeared to converge more slowly (Fig. S5 and S6, ESI†) and so this bilayer was simulated for twice as long, *i.e.* 10 μs. The fit of eqn (2) at low-*q* was less good than the Aqp0 simulation (Fig. S6, ESI†) and the value of the bending rigidity was predicted to be 16.1 ± 0.7*k*_B_ *T*, a significant reduction of 48% compared to the control POPC bilayer (Table 1). There is some evidence that a small shoulder is introduced into the power spectrum by the addition of the protein – this is more clearly seen in the graphs of *q* ^4^〈|*h*(**q**)|^2^〉 against *q* (Fig. S6, ESI†).

To the two-component POPE/POPG bilayer we inserted two different bacterial outer membrane proteins: the vitamin B_12_ transporter, BtuB, and the larger, trimeric porin, OmpF. Either 144 copies of BtuB, 100 copies of OmpF or 72 copies of each were inserted into the POPE/POPG bilayer and then simulated for 10 μs. These correspond to the protein occupying 28, 37 and 40% respectively of the area. Adding BtuB significantly decreased the stiffness of the bilayer, relative to the POPE/POPG lipid bilayer control, as shown by the value of *K*_c_ of 10.2 ± 1.1*k*_B_ *T*, a reduction of 52%. By contrast, adding OmpF at a higher concentration had no significant effect on the stiffness, with *K*_c_ = 19.4 ± 2.2*k*_B_ *T*. Examining more closely the graph of *q* ^4^〈|*h*(**q**)|^2^〉 against *q* (Fig. S6, ESI†) suggests that this system may not have reached the *q* ^4^ regime, and therefore an even larger simulation may be required to accurately predict the bending rigidity for this protein. Adding copies of both proteins has an intermediate effect with *K*_c_ = 16.6 ± 1.2*k*_B_ *T*, a 22% reduction in bending rigidity.

Although there is some evidence of additional features in the power spectra at intermediate values of *q*, these are not as pronounced as the ‘shoulders’ observed in the Kir2.2, and especially the Aqp0, simulations. Also, with the exception of Kir2.2, adding any of the integral membrane proteins to the lipid bilayers leads to a maximum in the power spectrum of the thickness fluctuations (Fig. S7, ESI†) that cannot be explained by eqn (4). By inspection, the proteins appear to be forming clusters on the tens of microseconds timescale (see the ESI,† Movies). To assess if these features in the power spectra are therefore due to protein clustering,^66^ we calculated the radial distribution functions (Fig. S8, ESI†). These show that the peripheral membrane protein, tN-Ras, does not aggregate in either ternary lipid mixture whilst all four integral membrane proteins cluster to a varying degree (see also the ESI,† Movies). BtuB and OmpF have a strong tendency to cluster but have the smallest ‘shoulders’ in the power spectrum of the height fluctuations, whilst the proteins with the largest ‘shoulders’, Kir2.2 and Aqp0, have not formed large clusters by the end of the simulations. Likewise, there is no correlation between the observed maxima in the power spectra of the thickness fluctuations and the tendency of any of the proteins to aggregate. We conclude that these features are not obviously due to protein clustering and that additional simulations, and possibly also new theory, will be required to explain them. One possible candidate theory couples the height and thickness fluctuations, introducing additional elastic constants.^52^

### 2.6 Adding peripheral membrane proteins does not alter stiffness

All the proteins studied thus far are integral membrane proteins; we shall now investigate the effect of adding a simple peripheral membrane protein, the truncated form of the cell signalling protein N-Ras, to the surface of the membrane. The Ras proteins segregate into distinct domains on the cell membrane,^67^ which are thought to correspond to disordered and ordered phases.^68^ Adding 108 copies of tN-Ras, which is uncharged, to one leaflet of either the low (Fig. 5C) or high cholesterol (Fig. 5D) mixtures has no effect on the power spectra of either the height or thickness fluctuations. This might seem surprising, however tN-Ras is only bound to the membrane by two lipids: a farnesyl and a palmitoyl which are covalently attached to two cysteines at the C-terminus of the protein. Unlike the integral membrane proteins, tN-Ras therefore minimally perturbs the bilayer. Since there is no significant effect, both power spectra are well-described by HC theory (Fig. S7, ESI†) and *K*_c_ is predicted to be 23.8 ± 2.2*k*_B_ *T* and 51.1 ± 2.9*k*_B_ *T* for the high- and low-cholesterol mixtures, respectively (Table 1 and Table S1, ESI†). Both these values lie within error of their respective pure bilayer control simulations. This is in agreement with an experimental study on small farnesylated peptides that demonstrated little or no effect on the bending rigidity.^69^

## 3 Discussion

Understanding how biological membranes can bend is essential for understanding a wide range of processes. Helfrich–Canham (HC) theory states that the key parameter for describing the stiffness of a biological membrane is the bending rigidity, *K*_c_. It is important to accurately measure *K*_c_ since thermally activated processes will depend exponentially on its magnitude and so small changes can have substantial effects.^70^ Here we have used molecular dynamics simulations of large lipid bilayers to investigate how either including membrane proteins or altering the lipid composition (including increasing the concentration of cholesterol) affect the bending rigidity. As expected, altering the composition of the lipid bilayer changed the bending rigidity. A ternary lipid mixture containing a small concentration of cholesterol (10%) was less rigid than a pure POPC bilayer, however, increasing the concentration of cholesterol to 60% (an unphysiologically high concentration) doubled the magnitude of the bending rigidity. These observations are consistent with experiments that varied the concentration of cholesterol and measured *K*_c_.^63,64^

Including integral membrane proteins tended to either have no significant effect or reduce the stiffness of the membrane, consistent with the currently limited experimental data on integral membrane proteins.^16,17^ The exception was the aquaporin, Aqp0, which was predicted to slightly increase the bending rigidity. Videomicroscopy experiments of bacteriorhodopsin have demonstrated that activating this protein increased the magnitude of fluctuations in the low-*q* region.^18^ Measurements of the bending rigidity as a function of the concentration of different Sar1 proteins also suggest that, at the right concentration, Sar1A may increase the bending rigidity.^10^ HC theory assumes the membrane can be described as an elastic sheet and therefore it is not clear to what extent the theory accommodates the effect of the addition of discrete inclusions, such as membrane proteins. The potential inability of HC theory to capture the effects of membrane proteins could be the origin of the unusual features in the power spectra we observed – we shall discuss this shortly.

**Fig. 6:**
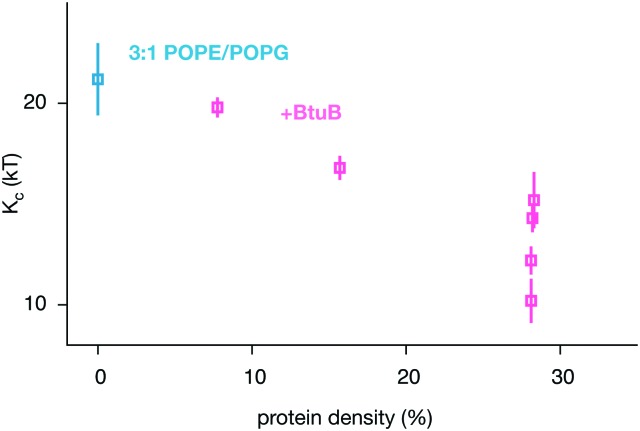
The bending rigidity decreases as the proportion of the area occupied by the integral membrane protein BtuB increases for the two component POPE/POPG bilayer. The additional BtuB datapoints come from a published series of coarse-grained simulations of smaller patches of POPE/POPG containing a range of densities of BtuB, OmpF and BtuB + OmpF66 – the results for all three protein combinations are given in Fig. S13 (ESI†). These simulations were analysed (Fig. S9–S12, ESI†) and added to our dataset to improve the statistics. Details of these simulations can be found in Table S2 (ESI†).

By combining our data with some previously published simulations,^66^ we can predict how varying the density of BtuB, OmpF or both proteins together in POPE/POPG bilayers affects the bending rigidity (Table 1 and Table S1, S2, ESI†). For BtuB, for which we have the largest dataset, the higher the protein density, the greater the reduction in the bending rigidity, *K*_c_ (Fig. 6) – this appears approximately linear based on our limited data. At a physiological protein density of ∼25%^1,2^ the bending rigidity was reduced by 25–50%. Adding small peptides to lipid bilayers have a concentration dependent effect, reducing the bending rigidity by as much as a factor of 2–10× at very high concentrations.^11–14^ By contrast, varying the density of OmpF had a relatively small effect on the bending rigidity (Fig. S13B, ESI†). Increasing the density of both proteins (when present in equal amounts) reduced the magnitude of the bending rigidity, *K*_c_ (Fig. S13C, ESI†), albeit not at the same rate as when only BtuB is inserted. That BtuB and OmpF, despite both being outer membrane proteins and therefore having similar shapes, have different effects on the rigidity of the lipid bilayer is significant since it has been suggested that the reduction in *K*_c_ observed for SERCA1A was due to that protein's inherent conical shape.^17^ Yet here we have two β-barrel proteins which are not conical, yet one has a markedly effect on the bending rigidity and the other does not – the main difference is that OmpF is a trimer and BtuB is a monomer, resulting in a *ca.* 2× difference in cross-sectional area. Studies of the trafficking protein Sar1 have shown that even different orthologs can have different effects on the bending rigidity of a lipid bilayer; adding increasing amounts of the yeast ortholog causes a large decrease in *K*_c_ whereas one of the two human paralogs has no or little effect whilst adding the other again leads to moderate reduction in the bending rigidity.^10^

**Fig. 7:**
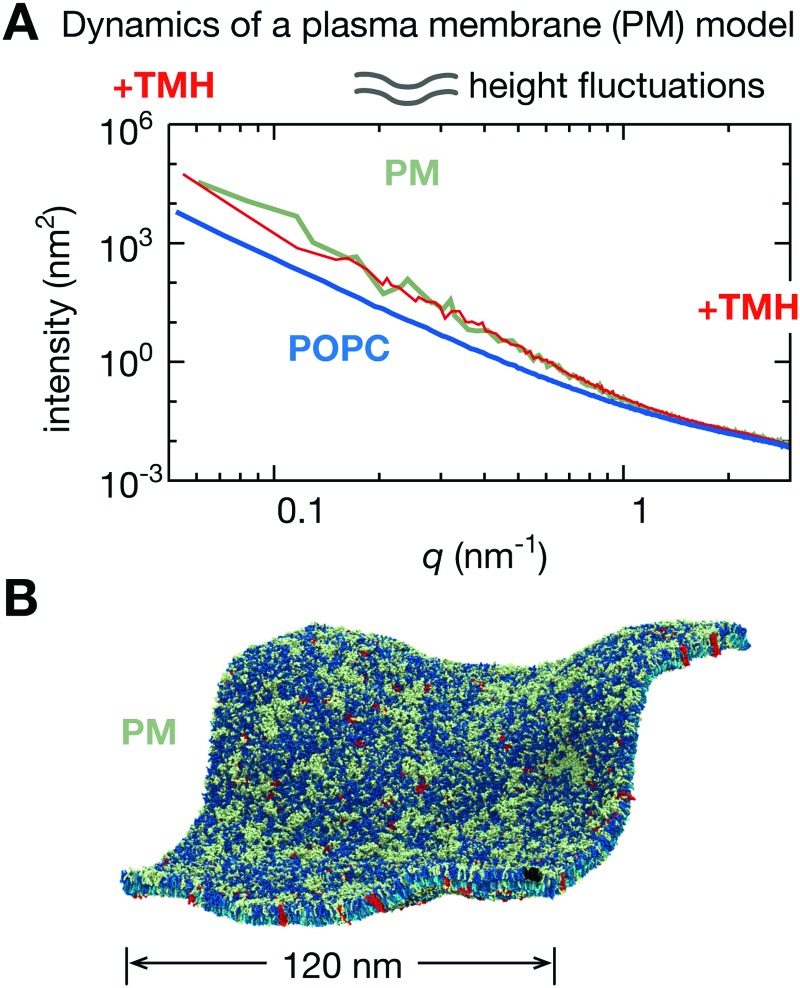
A more realistic model of the plasma membrane has large fluctuations in both height and thickness. (A) Inserting 576 copies of the transmembrane helix (TMH) of the gp130 cytokine receptor reduces the intensity of both the height and thickness power spectra. Since this is a log–log plot, this indicates the plasma membrane model is significantly less rigid as the pure POPC bilayer. (B) An image of the bilayer containing the transmembrane helix (in red) illustrating the large degree of curvature. We note that this bilayer, since it is becoming highly curved, can no longer be viewed as a perturbation from a flat sheet and therefore using the Monge gauge, *h*(*x*, *y*), to describe the height fluctuations, as done here, will become less appropriate.

What about more realistic models of biological membranes, in terms of lipid composition? Compared to our simple POPC bilayer, a plasma membrane model with seven different lipid species (including cholesterol) asymmetrically distributed between the two bilayer leaflets^40^ is ‘softer’ with larger fluctuations in the height power spectrum at low-*q* (Fig. 7). Inserting transmembrane helices into the plasma membrane model has no significant effect.

We have noted where Helfrich–Canham theory cannot explain all the dynamical effects we have observed; in particular adding integral membrane proteins, with the possible exception of BtuB, led to a ‘shoulder’ in the power spectrum of the height fluctuations at an intermediate value of *q* (Fig. 5 and Fig. S6, ESI†). This was especially pronounced for the aquaporin, Aqp0. Such features cannot be explained by the recent ‘tilt-dependent’ theory.^47–49^ Although we have not dwelled on the height fluctuations since we are primarily concerned with the stiffness of the membranes, we did observe instances where the power spectrum of the height fluctuations was not well described by eqn (4). In the atomistic simulation of the small POPC bilayer, rather than approaching an asymptote, the intensities at low-*q* appeared to be declining (Fig. 2). This could be an identical effect to the maximum we often observed at intermediate values of *q* in the much larger coarse-grained simulations (Fig. S7, ESI†), however, since the MARTINI coarse-grained forcefield was unable to reproduce the thickness power spectrum of the atomistic simulation, the thickness power spectra produced for the large coarse-grained lipid bilayers should be treated with caution. There are more complex theories that could potentially describe some of this behaviour seen here.^52^ Since the value of the bending rigidity is somewhat dependent on the underlying theory, we suggest that in more complex systems, such those including membrane proteins, it is desirable to report both the estimated bending rigidity and the experimentally measured power spectrum, where possible.

All of this suggests that the interplay between integral membrane proteins and lipids that governs the fluctuation dynamics of a membrane is rather complex. This is perhaps unsurprisingly since integral membrane proteins can not only perturb the local environment but also interact both directly^66,71^ and indirectly^72,73^ with a wide range of lipid species and other proteins, leading to clustering of proteins and/or lipids (see the ESI,† Movies). Thus in a crowded membrane, it is likely that the global stiffness depends not only upon the intrinsic elastic properties of both the protein and lipid components but also how they interact with each other. Early theoretical studies ignored the interactions between protein and lipid.^28^ These interactions will also affect the rate at which both proteins and lipids diffuse in the bilayer,^74,75^ altering the timescales of the dynamics. We note that, in each lipid bilayer, the protein that forms the largest clusters (Fig. S8, ESI†) by the end of the simulation (Kir2.2 in POPC and BtuB in POPE/POPG) is also the membrane where the bending rigidity is most affected (Fig. 5). Although speculative, this is also consistent with our earlier observation that the bending rigidity decreases approximately linearly with the concentration of BtuB (Fig. 6). Investigating these effects in detail and how they interact with one another to alter the bending rigidity of cell membranes will be the focus of future work.

## 4 Methods

### Creating the lipid bilayers

A bilayer of 1500 coarse-grained POPC lipids was self-assembled, as described elsewhere.^76^ An atomistic conformation was then created using a fragment-based approach.^77^ The ternary DOPC/sphingomyelin/cholesterol mixtures were created by ‘mutating’ the beads of the lipids *in situ.* ^40^ These were then tessellated onto a 6 × 6 grid to create the large ternary bilayers. A self-assembly simulation containing a single copy of Kir2.2^45^ along with POPC lipids was run.^76^ The resulting conformation was then tessellated onto a 12 × 12 grid creating an initial conformation for the large Kir2.2/POPC simulation. The large POPE/POPG membranes, including those containing BtuB and OmpF, were created by tessellating some previously published simulations.^66^ A truncated form of N-Ras^78^ was embedded by gradually ‘turning on’ a soft-core van der Waals potential between all copies of the protein and the lipids.^79^ Likewise, 144 copies of Aqp0^44^ were embedded in the large control POPC bilayer by the same process, which we call Alchembed.^79^ Sufficient water and ion beads were added in each case to allow room for the bilayer to fluctuate along the membrane normal. Details of each of the simulations can be found in Table S1 (ESI†). The plasma membrane models were constructed as described elsewhere.^41^

### Simulation parameters

The energy of the initial atomistic conformation was first minimised by the GROMACS molecular dynamics package^80^ using the steepest descent method. The temperature of the atomistic POPC system (Sim 1 – Table S1, ESI†) was gradually increased from 100 K to 310 K in 20 K steps, with 40 ps of molecular dynamics run at each step using a 2 fs timestep. Following this the dynamics of the system were simulated for 0.5 μs using GROMACS. Electrostatic forces were calculated between all atoms using the Particle Mesh Ewald method^81^ with a real space cutoff of 1.35 nm. van der Waals forces were calculated between all atoms separated by less than 1.35 nm. A Langevin thermostat with a time constant of 2 ps was used. Pressure was held at 1 bar by a semi-isotropic Berendsen barostat with a time constant of 1 ps and a compressibility of 4.46 × 10^–5^ bar^–1^. The lengths of all bonds involving a hydrogen were constrained using LINCS.^82^ Frames were saved to disc every 10 ps.

The coarse-grained simulations of pure lipid bilayers were run for 5 μs, whereas those containing proteins were run for 10 μs to ensure convergence, with the exception of those containing Aqp0 or tN-Ras. A timestep of 20 fs was used; this was reduced to 12 fs when cholesterol was present. This larger integration timestep is permitted by the coarse-graining and, along with reduced number of beads, and meant that the coarse-grained simulation of 1500 POPC lipid required ∼230× less computer resource than the fully-atomistic simulation. As is standard for MARTINI, electrostatic forces were calculated using a reaction field potential with a cutoff at 1.2 nm. van der Waals interactions were calculated between all beads separated by less than 1.2 nm, with the potential switched from 0.9 nm. The Verlet cutoff scheme was used. The temperature of the system was maintained using either a velocity rescale thermostat (sims 2, 3, 10–13) with a time constant of 1 ps or a Berendsen thermostat (sims 4–9) with a time constant of 4 ps. Both thermostats were coupled separately to proteins (if present), lipids and solvent. All simulations were run at 323 K, with the exception of the POPE/POPG simulations (sims 6–9) which were run at 313 K. The pressure was held at 1 bar using a semi-isotropic Berendsen barostat with either a time constant of 2 ps and a compressibility of 3 × 10^–4^ bar^–1^ (sims 2, 10–13) or a time constant of 4 ps and a compressibility of 5 × 10^–6^ bar^–1^ (sims 4–9). The exception was the large POPC bilayer simulation (sim 3) which used a semi-isotropic Parinello-Rahman barostat with a time constant of 12 ps and a compressibility of 3 × 10^–4^ bar^–1^. Coordinates were saved to disc every 200–600 ps.

### Analysis

The trajectories were first processed to ensure that there were no defects resulting from wrapping lipids in the periodic simulation box. All subsequent analysis was carried out using NumPy 1.9.1, SciPy 0.14.0 and MDAnalysis 0.8.1 in python 2.7.8.^83,84^ The surface of the bilayer was defined by the position of the phosphate beads and the height and thickness of the bilayer was interpolated onto a grid of size 0.5 nm using cubic splines. The resulting arrays were transformed into Fourier space using the FFTW routines present in the SciPy python module. The 1D power spectra were calculated by performing radial averaging. The current version of the code can be obtained from GitHub‖
‖https://github.com/philipwfowler/calculate-bilayer-power-spectrum
 and includes a simple worked example that reproduces the spectra shown in Fig. 2B and C. Graphs were plotted using gnuplot 4.6. Curves were fitted using the least-squares method in the SciPy python module and checked against the least-squares method in gnuplot. The values of *K*_c_ were fitted by plotting eqn (2) and only considering points which appeared to lie on a horizontal line (*i.e.* therefore confirm to 1/*q* ^4^). For simplicity we set this to be *q* < 0.6, 0.4, 0.2 nm^–1^ for small, medium and large patches of lipid bilayer, respectively. All fits can be found in Fig. 2, 3 and Fig. S6 (ESI†). Each simulation was tested for convergence by dividing each trajectory into 1000 (or 100) bins of equal width; the height power spectrum was calculated for each bin and fitted as described above. The timeseries of *K*_c_ values was then plotted, allowing a qualitative estimate of when each simulations had equilibrated (Fig. S2 and S4, ESI†). To provide a more quantitative estimate the statistical inefficiency was calculated as a function of both the bin width and degree of reverse penetration into each *K*_c_ dataset^85^ (Fig. S3 and S5, ESI†).
