# Supplementary material for: Membrane stiffness is modified by integral membrane proteins
Source: Soft Matter. 2016 Aug 26;12(37):7792–803. doi: 10.1039/c6sm01186a (PMC5314686; doi:10.1039/c6sm01186a)
Supplement: Supplementary file 12 [file SM-012-C6SM01186A-s012.pdf]

Electronic Supplementary Material (ESI) for Soft Matter.
This journal is © The Royal Society of Chemistry 2016

README.md

This file describes how these movies relate to the simulations discussed in the paper. The nomenclature is

```
movie-lipids-protein.mov
```

e.g.

```
movie-popc-kir2.2.mov
```

is a movie of Kir2.2 in the large POPC bilayer (sim #4 in Table S2 of the Supplementary Information). The ternary mixtures of DOPC, Sphingomyelin and Cholesterol are labelled pcsmchol and then followed by the ratio e.g. pcsmchol–226 is the high cholesterol mixture (60%). The below table relates each movie name to Table S2 of the Supplement.

Sim #	Movie Name	Legend
3	movie-popc.mov	Movie of POPC lipid bilayer (54,684 lipids, blue). Duration = 5μs, interval between frames = 50ns, 12 frames per second. Waters not shown for clarity.
4	movie-popc-kir22.mov	Movie of POPC lipid bilayer (55,584 lipids, blue) containing 144 copies of the inward-rectifying ion channel, Kir2.2 (red) . Duration = 10μs, interval between frames = 50ns, 12 frames per second. Waters not shown for clarity.
5	movie-popc-aqp0.mov	Movie of POPC lipid bilayer (37,249 lipids, blue) containing 144 copies of the aquaporin, Aqp0 (salmon) . Duration = 5μs, interval between frames = 50ns, 12 frames per second. Waters not shown for clarity.
		Movie of 3:1 POPE/POPEG lipid bilayer

6	movie-popeg.mov	(41,472 lipids, medium blue and cyan, respectively). Duration = 5μs, interval between frames = 50ns, 12 frames per second. Waters not shown for clarity.
7	movie-popeg-btub.mov	Movie of 3:1 POPE/POPEG lipid bilayer (28,888 lipids, medium blue and cyan, respectively) containing 144 copies of BtuB (pink). Duration = 10μs, interval between frames = 50ns, 12 frames per second. Waters not shown for clarity.
8	movie-popeg-ompf.mov	Movie of 3:1 POPE/POPEG lipid bilayer (26,832 lipids, medium blue and cyan, respectively) containing 100 copies of OmpF (magenta). Duration = 10μs, interval between frames = 50ns, 12 frames per second. Waters not shown for clarity.
9	movie-popeg-btubompf.mov	Movie of 3:1 POPE/POPEG lipid bilayer (25,448 lipids, medium blue and cyan, respectively) containing 144 copies of BtuB (violet). Duration = 10μs, interval between frames = 50ns, 12 frames per second. Waters not shown for clarity.
10	movie-pcsmchol–811.mov	Movie of 8:1:1 DOPC/sphingomyelin/cholesterol lipid bilayer (53,964 lipids, cyan, blue/green and yellow, respectively). Duration = 5μs, interval between frames = 50ns, 12 frames per second. Waters not shown for clarity.
11	movie-pcsmchol–811-tnras.mov	Movie of 8:1:1 DOPC/sphingomyelin/cholesterol lipid bilayer (53,964 lipids, cyan, blue/green and yellow, respectively) containing 108 copies of tN-Ras (orange). Duration = 5μs, interval between frames = 50ns, 12 frames per second. Waters not shown for clarity.

12	movie-pcsmchol–226.mov	Movie of 2:2:6 DOPC/sphingomyelin/cholesterol lipid bilayer (53,964 lipids, cyan, blue/green and yellow, respectively). Duration = 5μs, interval between frames = 50ns, 12 frames per second. Waters not shown for clarity.
13	movie-pcsmchol–226-tnras.mov	Movie of 2:2:6 DOPC/sphingomyelin/cholesterol lipid bilayer (53,964 lipids, cyan, blue/green and yellow, respectively) containing 108 copies of tN-Ras (orange). Duration = 5μs, interval between frames = 50ns, 12 frames per second. Waters not shown for clarity.

All movies run at 12 frames per second with 50 ns between frames. They are not to scale.

Philip W Fowler, May 2016
