# Supplementary material for: Membrane stiffness is modified by integral membrane proteins
Source: Soft Matter. 2016 Aug 26;12(37):7792–803. doi: 10.1039/c6sm01186a (PMC5314686; doi:10.1039/c6sm01186a)
Supplement: Supplementary file 13 [file SM-012-C6SM01186A-s013.pdf]

Electronic Supplementary Material (ESI) for Soft Matter.
This journal is © The Royal Society of Chemistry 2016

Membrane Stiffness is Modified by Integral Membrane Proteins – Supporting Information.

Philip W Fowler[1,†], Jean Helie[1,‡], Anna Duncan[1], Matthieu Chavent[1], Heidi Koldsø[1,§], and Mark S P Sansom[1,*]

[1]Department of Biochemistry, University of Oxford, South Parks Rd, Oxford, OX1 3QU, UK

List of Figures

S1 Images of the proteins studied . . . 4
S2 Time series of K_c, K_d & K_e for the POPC bilayers . . . 5
S3 The statistical inefficiency of K_c, K_d & K_e for the POPC bilayers . . . 6
S4 Time series of K_c values for the large bilayers . . . 7
S5 The statistical inefficiency of K_c for the large bilayers . . . 8
S6 Determination of K_c for the large bilayers . . . 9
S7 The power spectrum of the thickness fluctuations of the large bilayers . . . 10
S8 All the integral membrane proteins show a prevalence to form clusters. . . . 11
S9 Determination of K_c for a series of published simulations . . . 12
S10 The calculated values of K_c for a series of published simulations . . . 13
S11 The power spectrum of the height fluctuations for a series of published simulations . . . 13
S12 The power spectrum of the thickness fluctuations for a series of published simulations . . . 14
S13 The bending rigidity of the membrane changes as the amount of protein added increases. . . . 15

List of Tables

S1 Details of the simulations studied in this work . . . 2
S2 Details of other published simulations that were analysed in this work . . . 3

*To whom correspondence should be addressed. Email: mark.sansom@bioch.ox.ac.uk
†Present address: Nuffield Department of Medicine, University of Oxford, John Radcliffe Hospital, Oxford, OX3 9DU
‡Present address: Semmle, Blue Boar Court, 9 Alfred St, Oxford OX1 4EH
§Present address: D. E. Shaw Research, New York, NY 10036

Sim no.	Lipids	No. Lipids	Forcefield	Protein	% Area	Width	No. particles	Duration	K_c (kT)
1	POPC	1,500	AT	–	–	22.0 nm	522,309	0.5 μs	29.8 $\pm$ 5.5
2		1,500	CG	–	–	22.3 nm	47,991	0.5 μs	25.2 $\pm$ 2.2
								5.0 μs	25.5 $\pm$ 0.5
3		54,684	CG	–	–	132 nm	2,053,296	5.0 μs	30.9 $\pm$ 1.3
4		37,249	CG	+144 Aqp0	29.4%	130 nm	2,165,000	5.0 μs	37.3 $\pm$ 3.6
5		55,584	CG	+144 Kir2.2	10.9%	142 nm	3,521,232	10.0 μs	16.1 $\pm$ 0.7
6	POPE/POPG 3:1	41,472	CG	–	–	111 nm	1,326,184	5.0 μs	21.2 $\pm$ 1.8
7		28,888	CG	+144 BtuB	28.1%	109 nm	1,331,636	10.0μs	10.2 $\pm$ 1.1
8		26,832	CG	+100 OmpF	36.8%	112 nm	1,370,636	10.0μs	19.4 $\pm$ 2.2
9		25,448	CG	+72 BtuB/72 OmpF	39.5%	112 nm	1,377,536	10.0μs	16.6 $\pm$ 1.2
10	DOPC/SM/CHOL 8:1:1	53,964	CG	–	–	128 nm	2,107,010	5.0 μs	25.0 $\pm$ 2.0
11		53,964	CG	+108 tN-Ras	0.2%	128 nm	2,108,954	5.0 μs	23.8 $\pm$ 2.2
12	DOPC/SM/CHOL 2:2:6	53,964	CG	–	–	99.1 nm	1,458,966	5.0 μs	49.1 $\pm$ 6.7
13		53,964	CG	+108 tN-Ras	0.3%	99.2 nm	1,458,966	5.0 μs	51.1 $\pm$ 2.9

Table S1: Details of the simulations studied in this work. The protein density was estimated by calculating the average area of the last 20% of each simulation and inferring the protein area using the appropriate area per lipid value calculated from the control simulations. Likewise the width reported is the average of the last 20% of each simulation. The effect of inserting half the proteins in one direction into the bilayer and the other half in the other direction was tested explicitly in the Aqp0 simulation labelled ‘symmetric’. The AT and CG forcefields used were CHARMM36[1] and MARTINI2.2[2,3], respectively.

Sim no.	Lipids	No. Lipids	CG Forcefield	Protein	% Area	Width	No. beads	Duration	K_c (kT)
14	POPE/POPG 3:1	2,528	Mixed	+1 BtuB	7.8%	28.4 nm	67,250	10.0 μs	19.8 $\pm$ 0.5
15		2,336	Mixed	+4 BtuB	15.7%	28.6 nm	61,813	10.0 μs	16.8 $\pm$ 0.6
16		2,012	Mixed	+9 BtuB	28.3%	28.8 nm	62,788	10.0 μs	15.2 $\pm$ 1.4
17		2,484	Mixed	+1 OmpF	10.3%	28.6 nm	67,540	10.0 μs	19.8 $\pm$ 0.4
18		2,238	Mixed	+2 BtuB/2 OmpF	20.7%	28.8 nm	68,221	10.0 μs	16.1 $\pm$ 0.5
19		1,842	Mixed	+5 BtuB/4 OmpF	37.2%	29.4 nm	69,636	10.0 μs	11.9 $\pm$ 0.8
20	POPE/POPG 3:1	8,052	Mixed	+36 BtuB	28.1%	57.5 nm	253,535	10.0 μs	12.2 $\pm$ 0.7
21		7,222	MARTINI	+36 BtuB	28.7%	54.7 nm	332,909	10.0 μs	14.3 $\pm$ 0.7

Table S2: Details of the simulations performed by Rassam *et al.*[4] that were analysed in this work. As before, the protein density was estimated by calculating the average area of the last 20% of each simulation and inferring the protein area using the appropriate area per lipid value calculated from the control simulations. The forcefield marked 'Mixed' represents the proteins using a coarse-grained forcefield[5] designed to work with an older version of MARTINI for the lipids and waters[6].

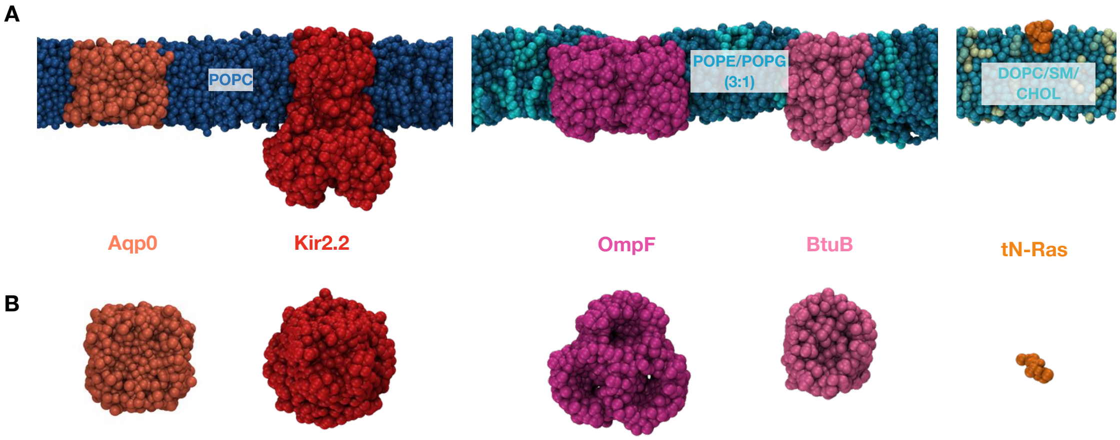

Figure S1: The effect of adding five membrane proteins was studied. Multiple copies of either an aquaporin, Aqp0[7], or an inward-rectifying potassium channel, Kir2.2[8], were added to a pure POPC lipid bilayer, as a simple model of a eukaryotic membrane. Two bacterial outer membrane proteins, the porin OmpF[9] and the vitamin B_{12} transporter BtuB[10], were added to the simple model of an *E. coli* membrane. This is composed of POPE and POPG in the ratio 3:1. Lastly, the truncated form of the cell-signalling kinases N-Ras was added to two different ternary mixtures of DOPC, Sphingomyelin and Cholesterol that are used to study the effect of lipid ordering and disordering[11]. Shown are (*A*) views in the plane of the membrane and (*B*) views from the extracellular medium (except in the case of tN-Ras where it is the intracellular medium).

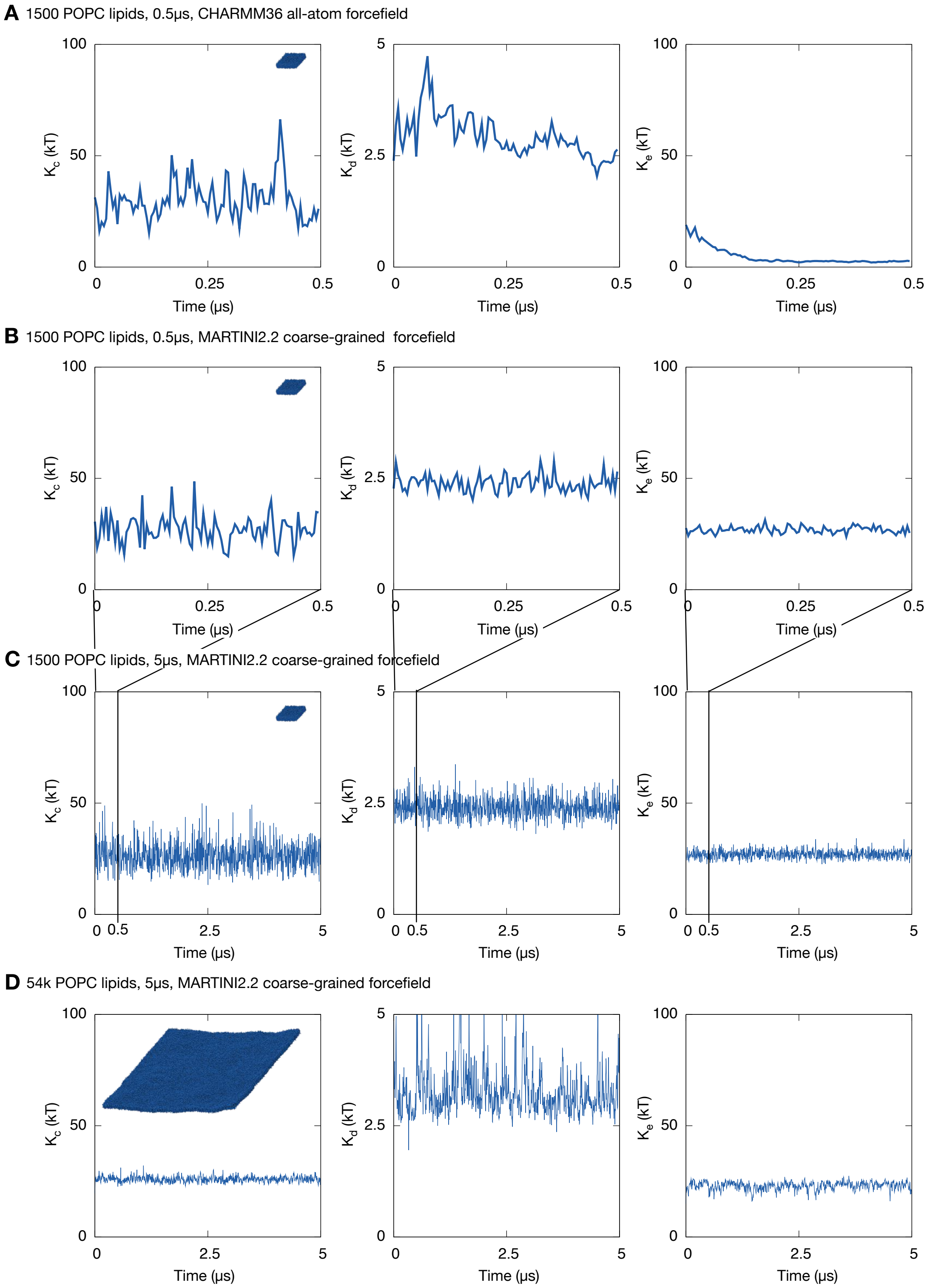

Figure S2: The convergence of the POPC simulations is first qualitatively assessed by examining how the values of K_c, K_d & K_e vary with time; if a simulation is converged these timeseries should be stationary. In all cases the width of a bin is 5 ns. (*A*) The height fluctuations (K_c) of a 1,500 POPC lipid bilayer modelled using an atomistic forcefield (CHARMM36) rapidly reaches equilibrium during a 0.5 μs simulation, whereas the thickness fluctuations (K_d, K_e) appear to require at least 0.2 μs to equilibrate. (*B*) All three timeseries derived from a 0.5 μs simulation of the same 1,500 POPC lipid bilayer, this time described using the MARTINI2.2 coarse-grained forcefield, fluctuate around an average value, suggesting that they have converged rapidly. (*C*) Extending the duration of the coarse-grained simulation by a factor of 10 does not reveal any longer time-scale dynamics. (*D*) The time-series of K_c for a much larger POPC bilayer (36x larger by area) fluctuates less; this is because K_c can be more accurately determined since lower-q modes are sampled. All three time series again suggest the simulation has rapidly converged.

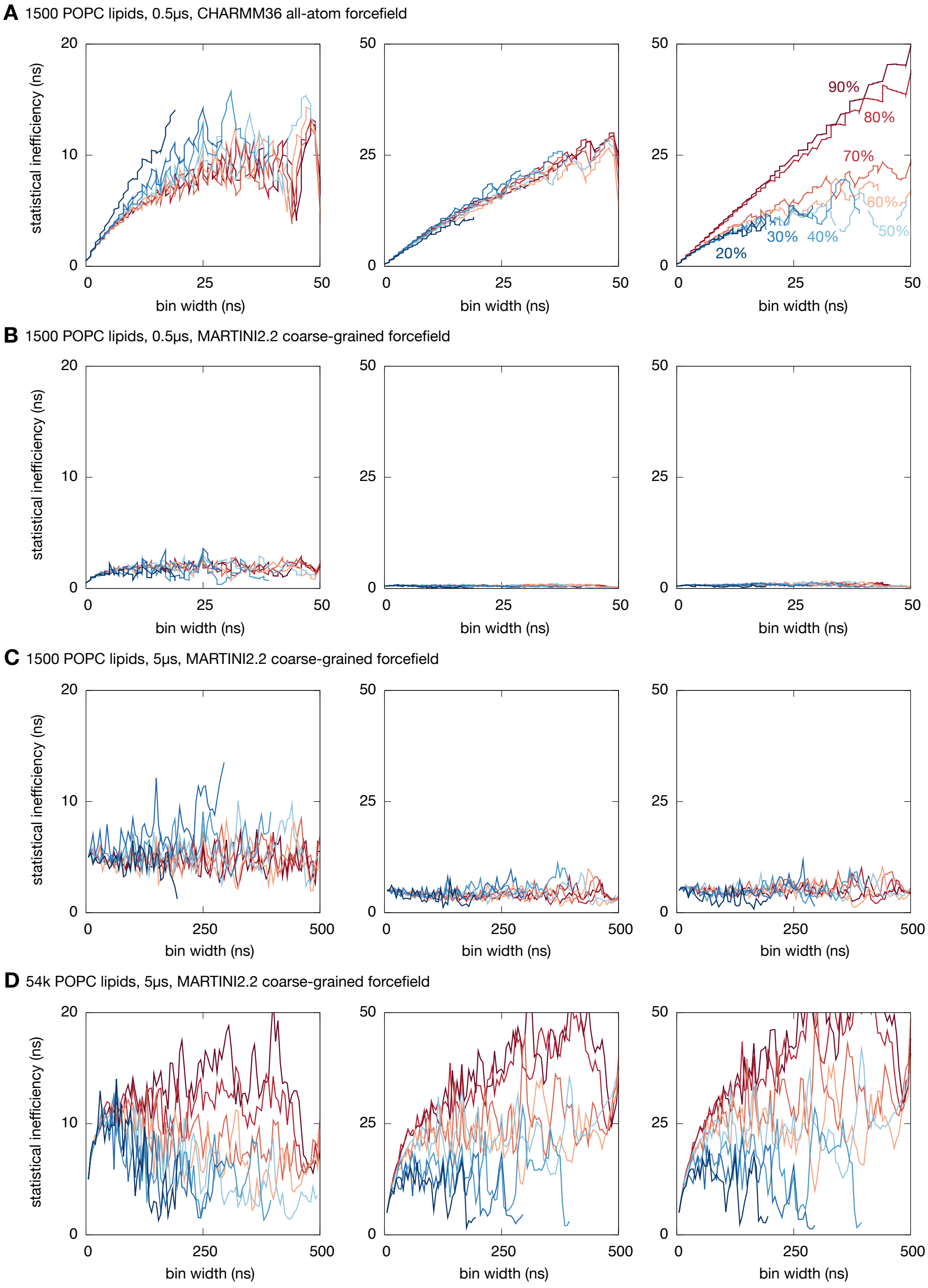

Figure S3: A more quantitative assessment of convergence is provided by calculating the statistical inefficiency for the datasets shown in Fig. S2. The statistical inefficiency of each series is calculated as a function of (1) the size of each bin and (2) how much of the data is included, starting from the end of each dataset. If the statistical efficiency approaches an asymptote then this indicates the data are stationary and is an estimate of the correlation time of the dataset[12]. If it does not, then the dataset is either not converged or is shorter than the correlation time. (*A*) The statistical inefficiency of the bending modulus, K_c, of 1,500 POPC lipids modelled using an atomistic (AT) forcefield (CHARMM36) approaches a plateau of 10 ns irrespective of the amount of data discarded. The picture for the thickness fluctuations is more complex: analysing K_d suggests the thickness fluctuations may not be converged, whereas the values of K_e appear to be converged if the first 40% of the data are discarded, yielding a correlation time of $\sim$ 20 ns. (*B*) Analysing the statistical inefficiency of the three elastic moduli calculated from the coarse-grained (CG) simulation of 1,500 POPC lipids suggest that all modes converge rapidly and the correlation time is $\sim$ 5-10 ns. (*C*) Repeating the analysis on the 10x longer dataset suggests that the first 20% should be discarded, giving a correlation time of $\sim$ 5 ns. (*D*) Repeating the analysis for the much larger 54,684 POPC CG lipid bilayer suggests $\sim$30% of the data should be discarded, leading to a correlation time of 10 ns for K_c and 25 ns for K_d and K_e.

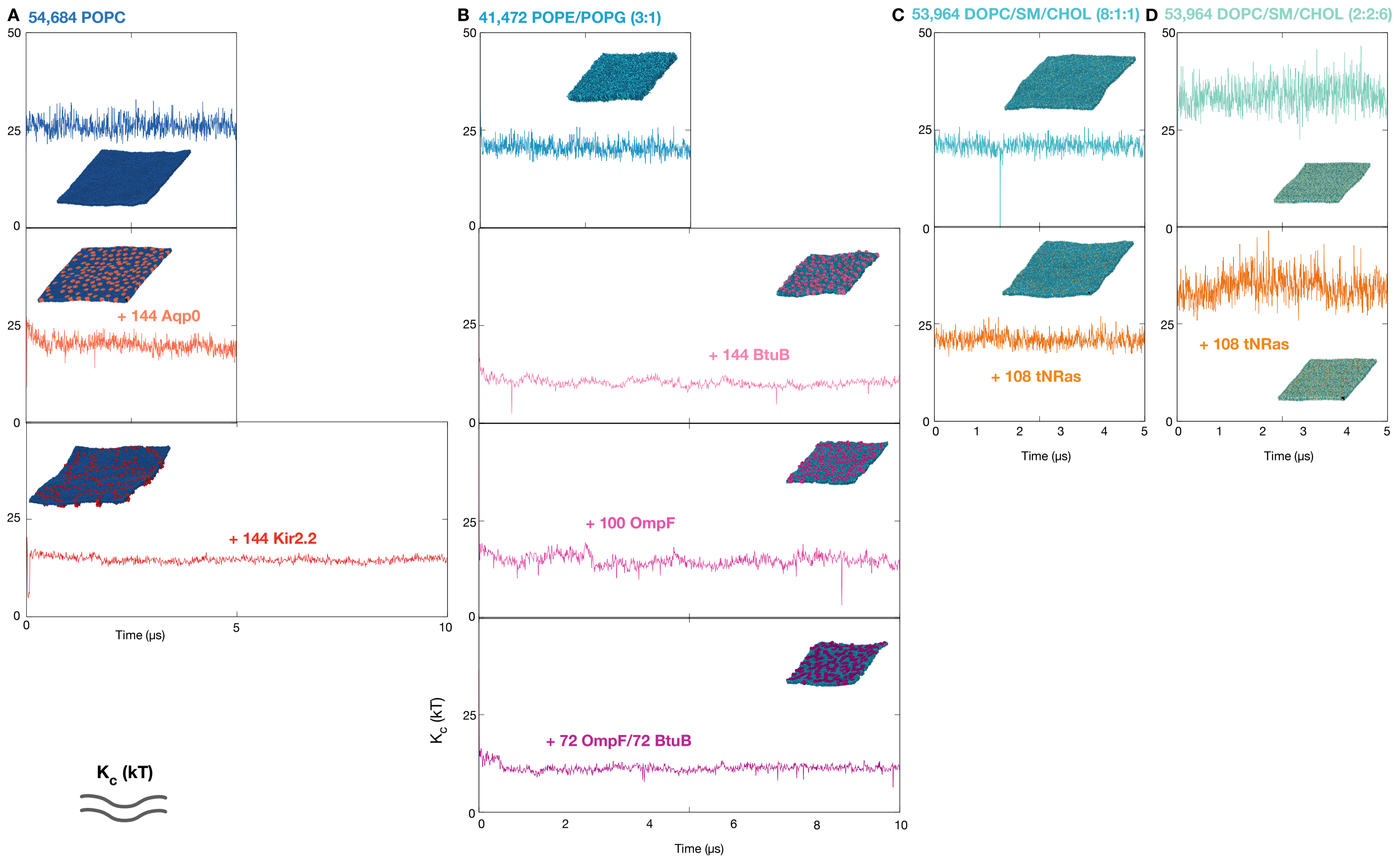

Figure S4: We now apply the same qualitative analysis of convergence shown in Fig. S2 to the large coarse-grained simulations. Each simulation is divided into 1000 bins and the value of K_c calculated for each bin. As before, visually examining the timeseries is a simple way of estimating convergence. Adding a protein appears, in some cases, to lead to an initial perturbation lasting up to 2 μs, suggesting that at least the first 2 μs of each trajectory should be discounted in any analysis.

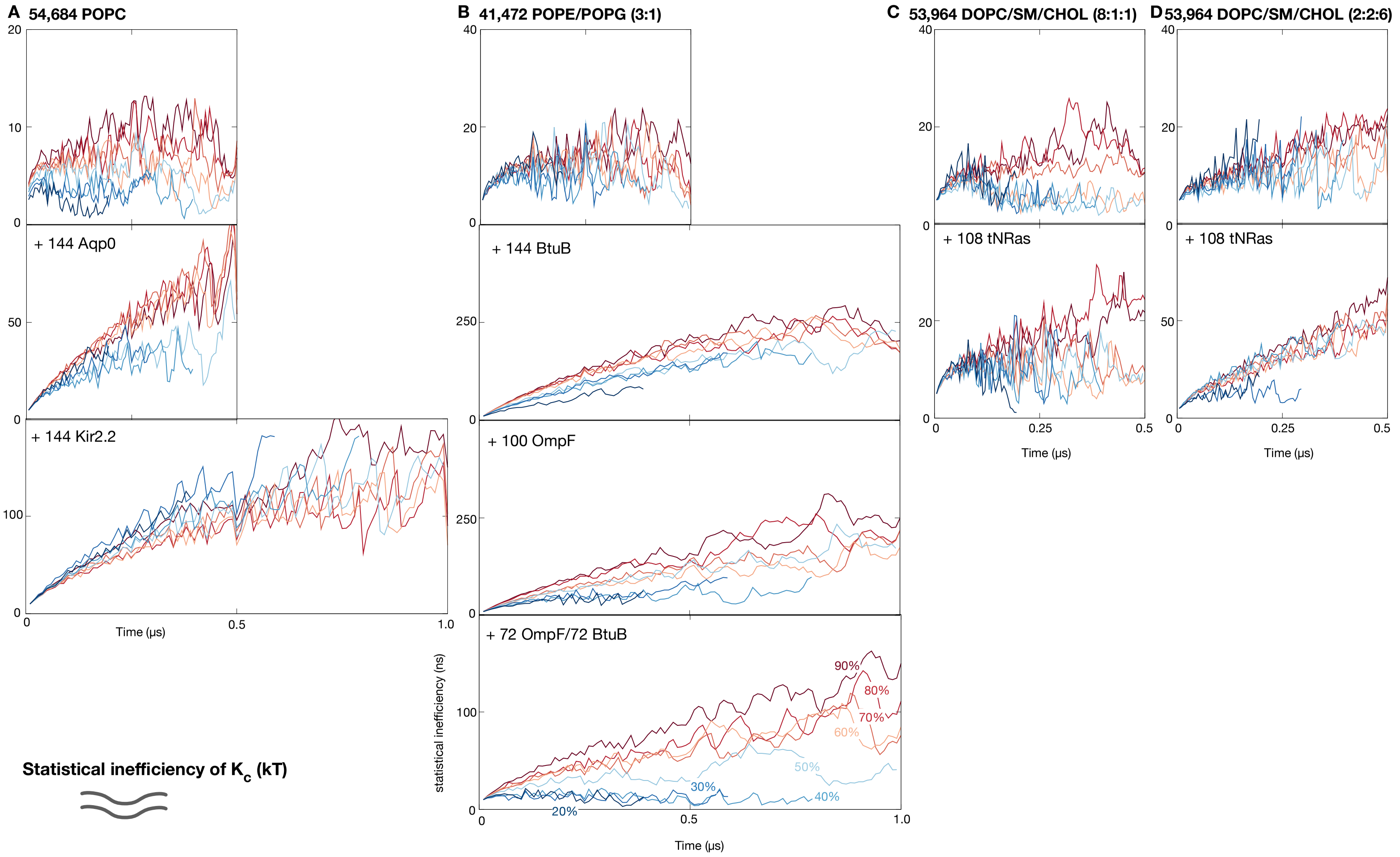

Figure S5: The statistical inefficiency is calculated for the data plotted in Fig. S4 as a function of both the bin width and the proportion of the dataset used, starting from the last point and moving towards the start. These plots either indicate that convergence is very rapid, or in some cases occurs in the first 30-50% of the trajectory. Estimated correlation times vary from 5 to 200 ns. We shall be conservative and discard the first 50% of each of these simulations and divide the remaining data can be divided into ten bins. The correlation time is therefore assumed to be 250 ns for 5 μs simulations and 500 ns for 10 μs simulations.

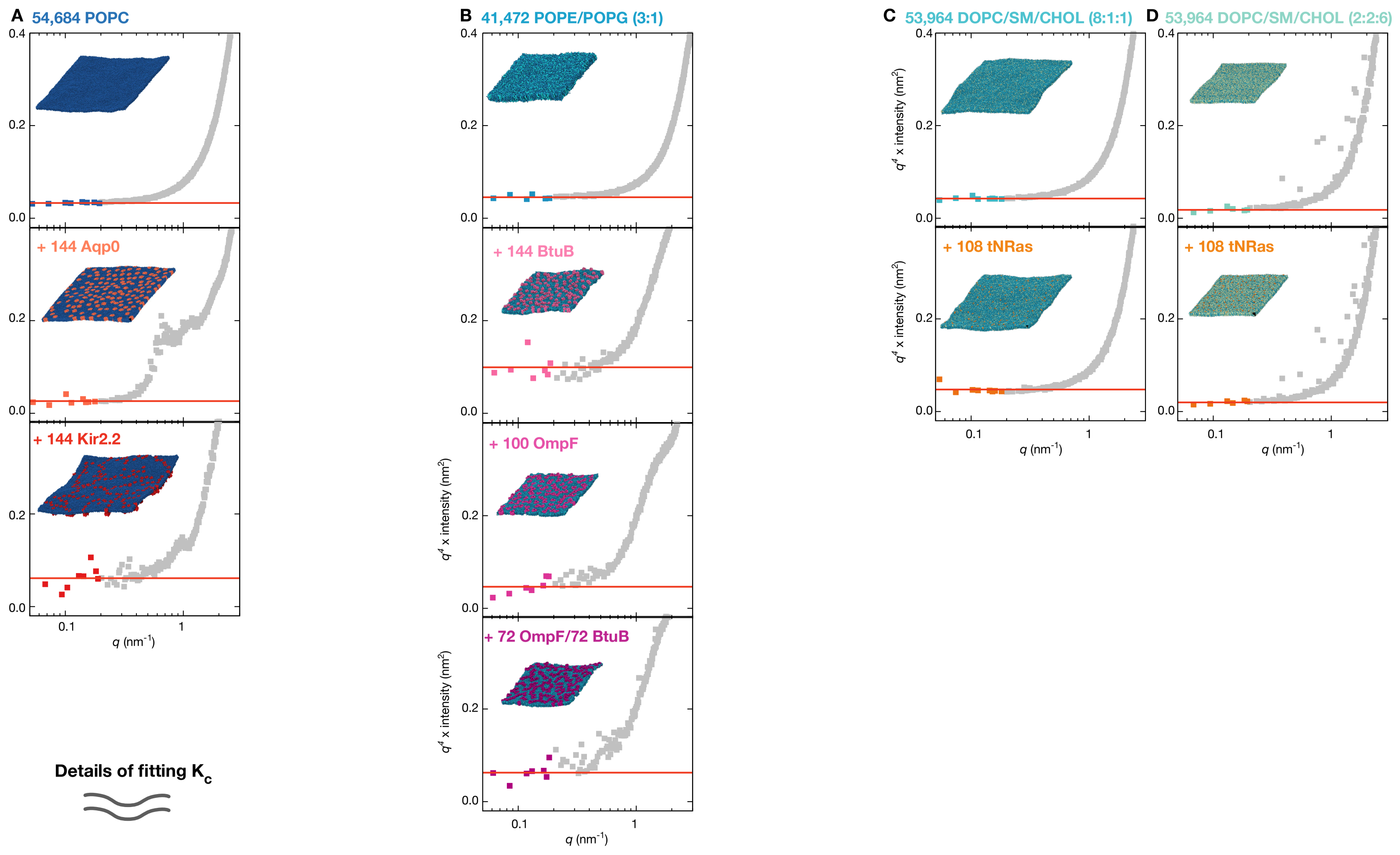

Figure S6: Helfrich-Canham theory predicts that a plot of $q^4\times$ the intensity of the power spectrum of the height fluctuations against q will be a constant. Fitting K_c using this relationship is more accurate since it gives each points the same weight. We have plotted this relationship for all the large bilayers studied. In all cases, a constant is approached as $q \to 0$, and in some cases there is complex behaviour at intermediate values of q, such as in the Aqp0 simulation. A constant is fitted to all points where $q < 0.2$ nm^{-1} – these points are coloured, with the others drawn in grey. The resulting fitted constant is drawn as a red line. These fits are then drawn in Figures 4 & 5 of the main body of the paper. In all cases, the first half of each simulation has been discarded. Note that the fit tends to be less good when protein is present; this may indicate that these simulations are not yet converged, or that, in these cases, still larger box sizes may be required to reach the Helfrich-Canham q^4 regime. Finally, the spurious points in the high cholesterol power spectra are likely due to difficulties in defining the surface of the lipid bilayer due to the high proportion of cholesterol (thereby reducing the area density of the phosphate beads).

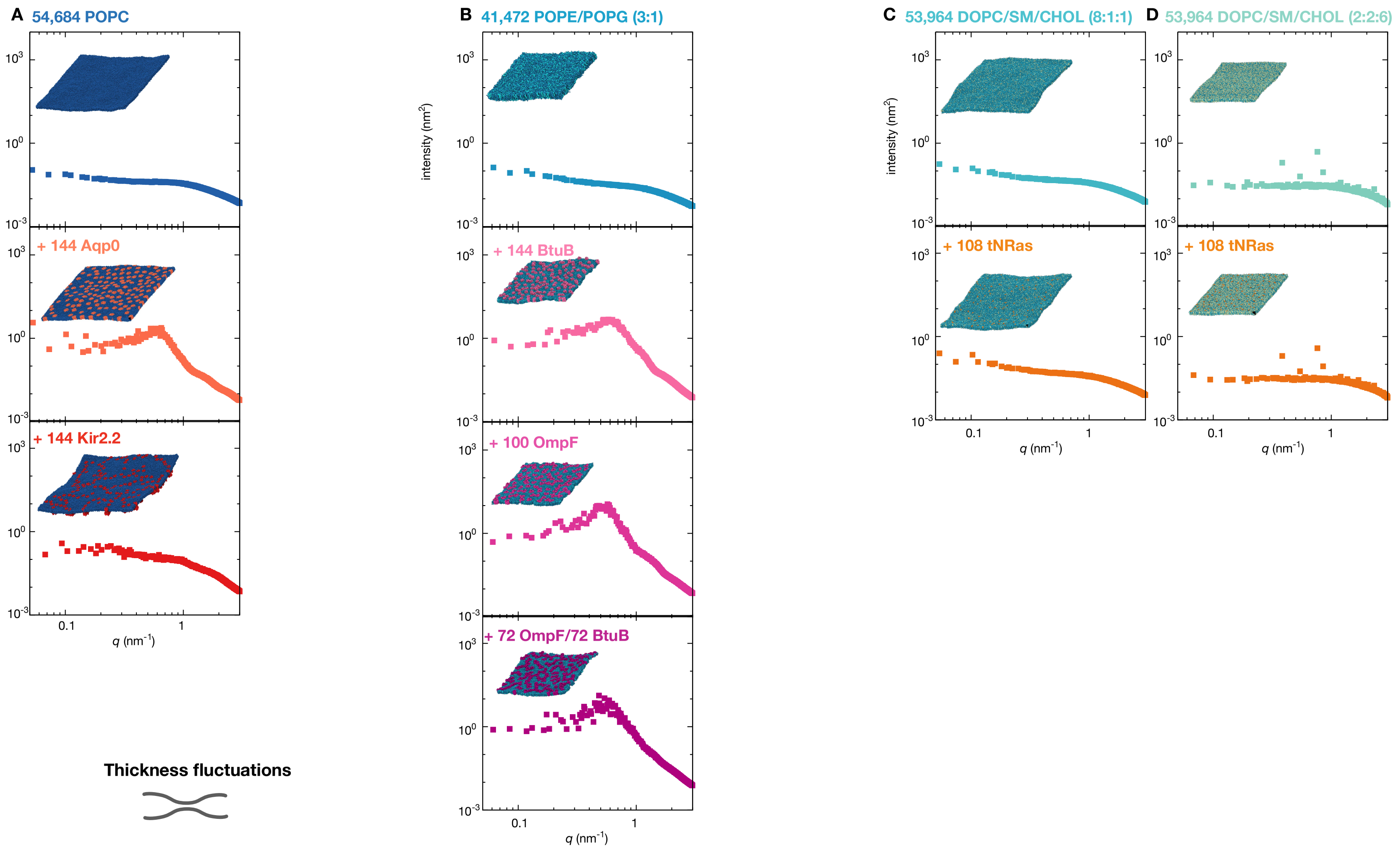

Figure S7: The power spectrum of the thickness fluctuations in the large coarse-grained bilayers deviate from Helfrich-Canham theory. The latter predicts that as $q \to 0$, the intensity should tend towards a constant, $k_b T / K_e$. Note that these are all log-log plots. (*A*) The intensity of the large POPC bilayer gradually increases as $q \to 0$. Adding 144 copies of Aqp0 introduces a maximum in the intensity at around $q \sim 0.6$ nm^{-1}. Adding 144 copies of Kir2.2 does not lead to such a maximum. (*B*) The intensity of the 3:1 POPE/POPG behaves similarly to the pure POPC bilayer and increases as $q \to 0$. In a similar fashion to the Aqp0/POPC simulation, adding BtuB, OmpF or both proteins to the POPE/POPG bilayer results in a maximum in the intensity at $q \sim 0.6$ nm^{-1}. The intensity of the (*C*) low cholesterol ternary mixture also increases as $q \to 0$, however the intensity of the (*D*) high cholesterol mixture does appear to asymptote at low q. There is little change upon adding the truncated form of the peripheral membrane protein, N-Ras.

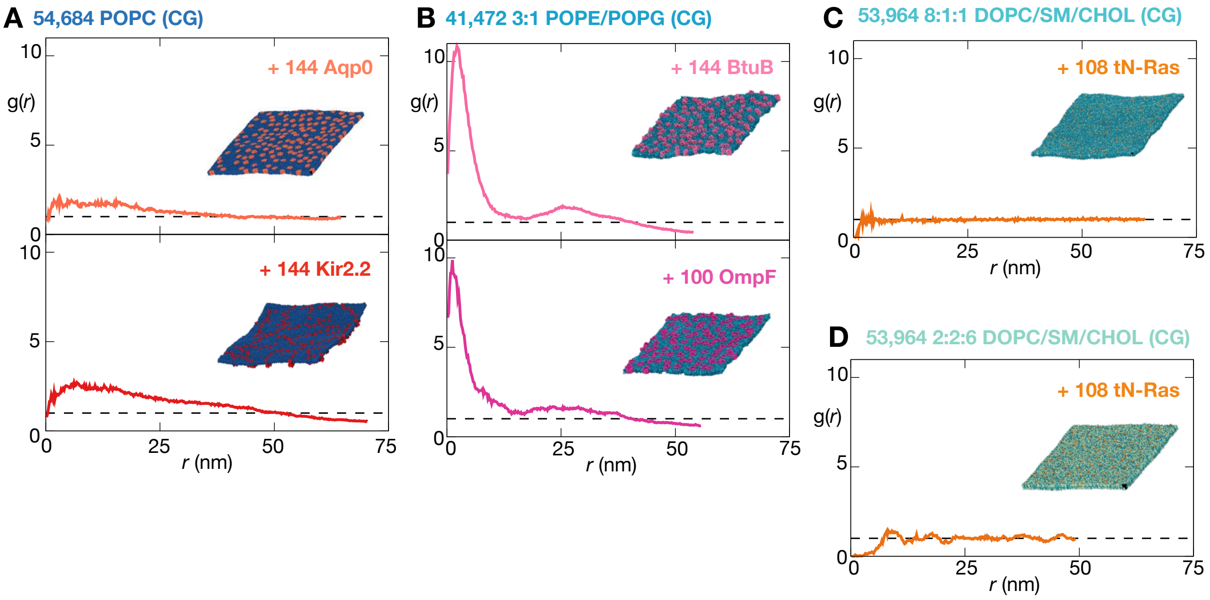

Figure S8: The radial distribution function for the protein center of mass – protein center of mass distances shows all the integral membrane proteins display a tendency to cluster during the simulations. (*A*) Both Kir2.2 and Aqp0 begin to demonstrate a moderate tendency in POPC to aggregate during the simulations, as indicated in the maximum in the radial distribution function, $g(r)$, at around a distance of 10-15 nm. (*B*) BtuB and OmpF have formed connected clusters in the POPE/POPG bilayer by the end of the simulations, as shown by both the pronounced maximum in the radial distribution function at around 5 nm, and the subsequent broader peak at around 25 nm. (*C*) By contrast, the peripheral membrane protein tN-Ras shows no tendency to aggregate in the low cholesterol ternary lipid mixture. (*D*) In the high cholesterol mixture, however, the radial distribution function has additional fluctuations, including a low probability region at small distances. These are due, we expect, to the lower rate of diffusion of tN-Ras in this lipid mixture, as can be seen in the Supplemental Movies. All radial distribution functions take into account the periodic boundary conditions and were calculated for the last 50% of each simulation.

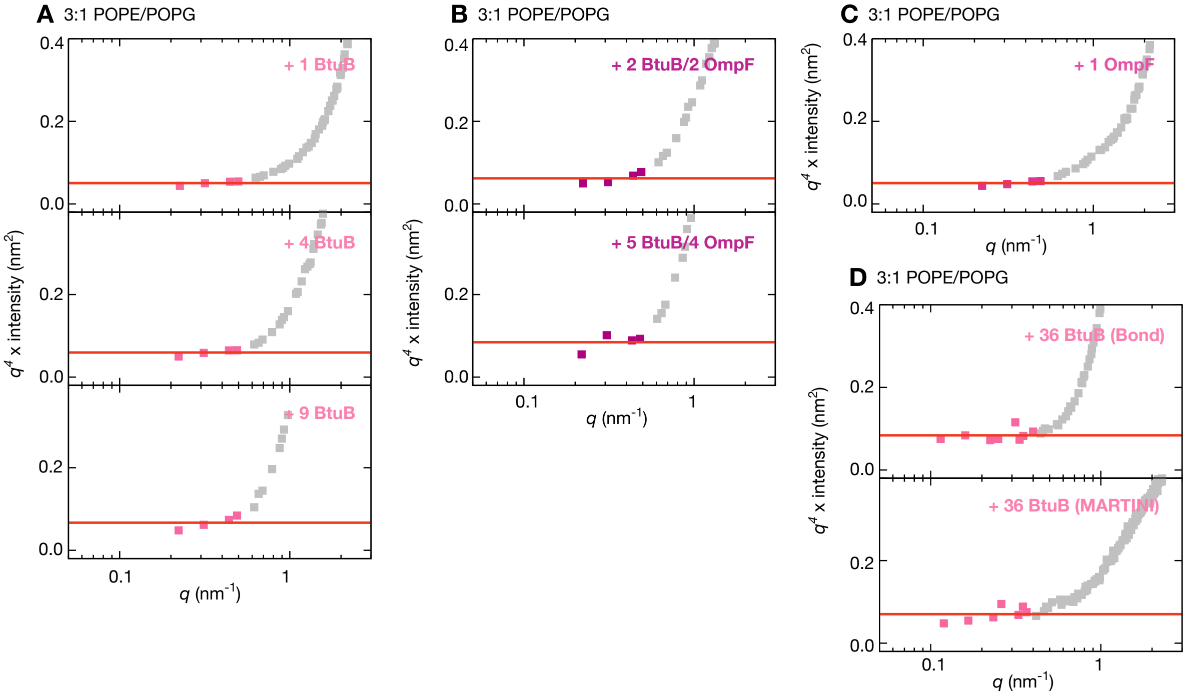

Figure S9: We follow the same procedure as in Fig. S6 to determine the values of K_c of a series of published simulations[4] of small (∼30 nm) and medium (∼55 nm) sized POPE/POPG 3:1 lipid bilayers containing varying numbers of BtuB and OmpF. Except where marked, all simulations represented the proteins using a coarse-grained forcefield[5] designed to work with an older version of MARTINI for the lipids and waters[6]. The first six simulations are small, having between 1842 and 2528 lipids, and therefore only intensities where $q < 0.6$ nm^{-1} are considered in the fit. As the last two simulations are somewhat larger, we were able to consider intensities where $q < 0.4$ nm^{-1}. (*A*) Increasing the number, and therefore density, of BtuB proteins in a small patch of POPE/POPG lipids worsens the correspondence with HC theory at low q. (*B*) A similar trend is seen when multiple copies of BtuB and OmpF are added to the same small patch of POPE/POPG lipids. Note that we were unable to analyse a third simulation with 8 copies each of BtuB and OmpF as the protein density was so high (68%) it became difficult to define and extrapolate the surface of each leaflet using only the positions of the lipid phosphates. (*C*) Only a single simulation with just OmpF was performed so no trend can be seen. (*D*) To assess the behaviour of the older forcefield[5], two patches of 7-8k lipids were simulated, one with the older forcefield and one with MARTINI[2,3]. By inspection the behaviour is similar.

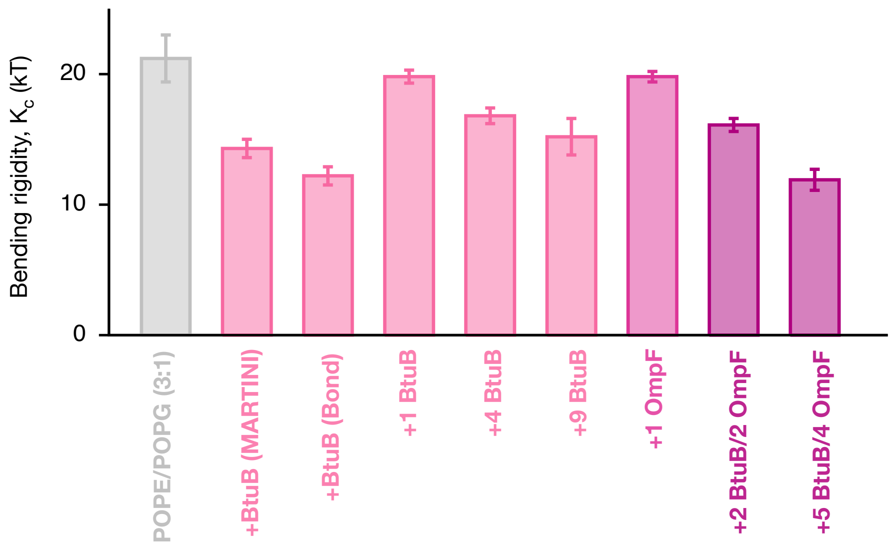

Figure S10: The calculated values of K_c for a series of published simulations[4] derived from the fits in Fig. S9. As per the large bilayers in the main body of the paper, the first half of each trajectory is discarded and errors are calculated by dividing the remainder into ten bins which are assumed to be independent. For a given protein, as its density is increased, the magnitude of the bending rigidity decreases.

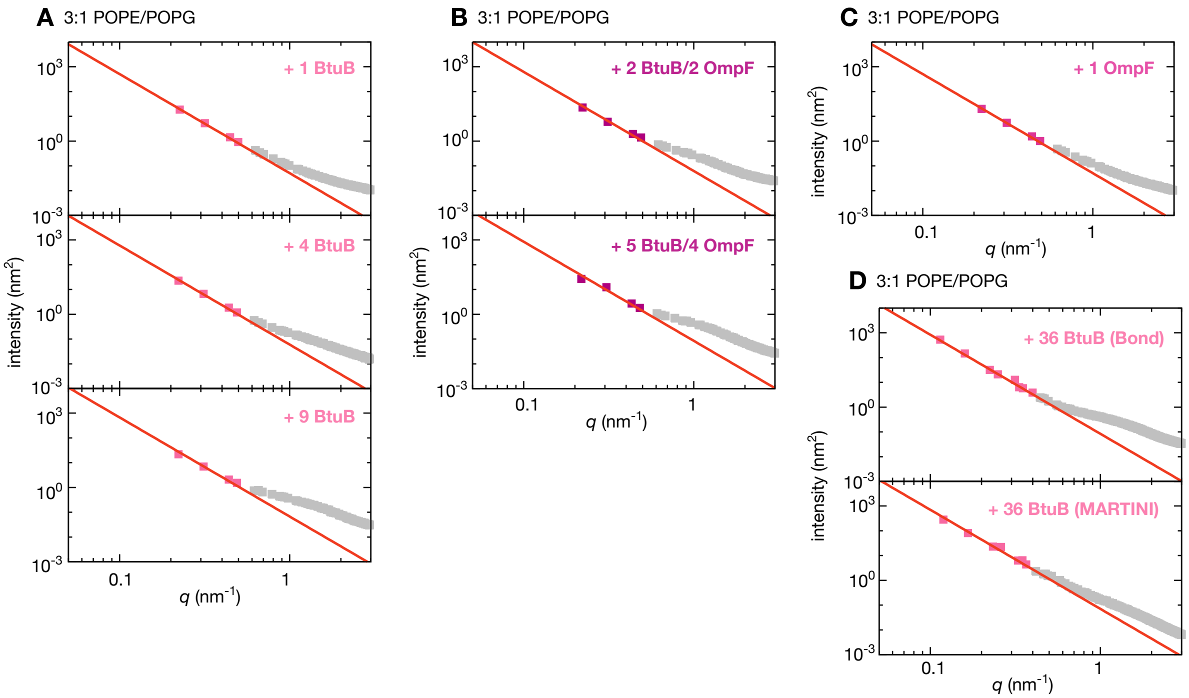

Figure S11: The power spectra of the height fluctuations for a series of published simulations[4]. All simulations appear to tend towards $1/q^4$ behaviour at low q, as predicted by Helfrich-Canham theory. Each fitted red line is derived from Fig. S6 and the points considered are drawn as coloured squares. Note that these are a log-log plots.

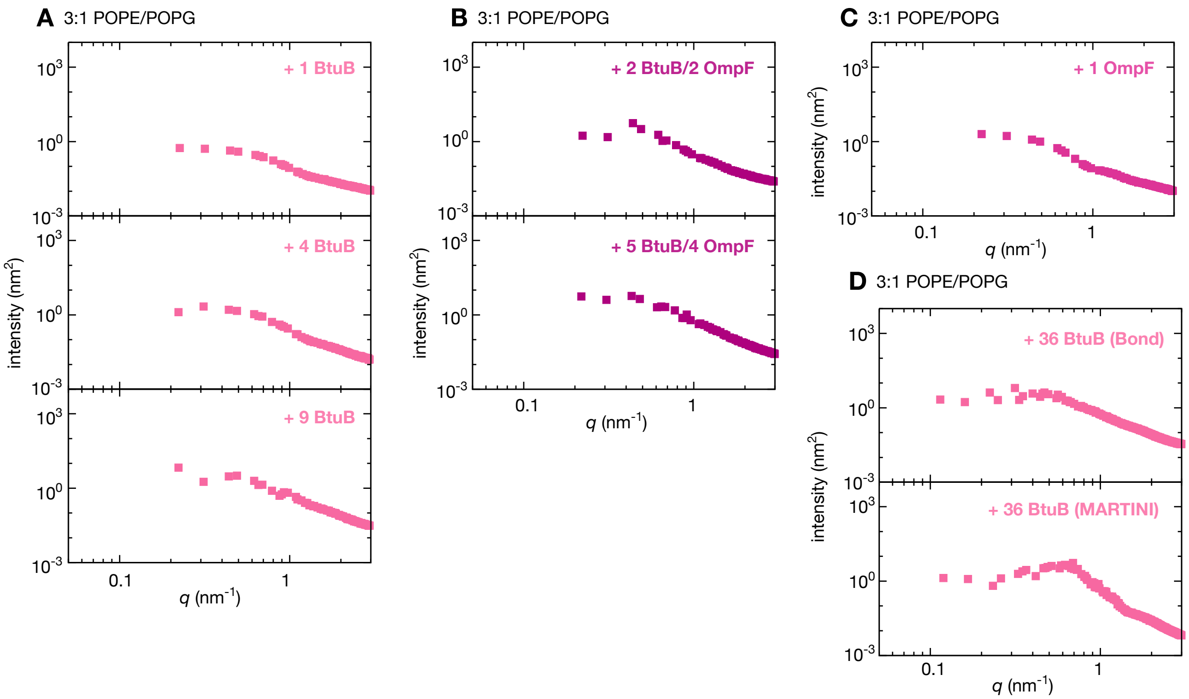

Figure S12: The power spectrum of the thickness fluctuations for a series of published simulations[4]. As these simulations are smaller than those in Fig. S7 small values of q are not sampled and so it is more difficult to determine if the intensity is approaching an asymptote as predicted by Helfrich-Canham theory. Increasing the number of (*A*) BtuB or (*B*) BtuB and OmpF proteins does not affect the power spectrum of the thickness fluctuations in a systematic manner. (*D*) There is a hint that the maximum, seen in the larger coarse-grained simulations (Fig. S7), is less pronounced when an older coarse-grained forcefield[5] is used. Note that these are log-log plots.

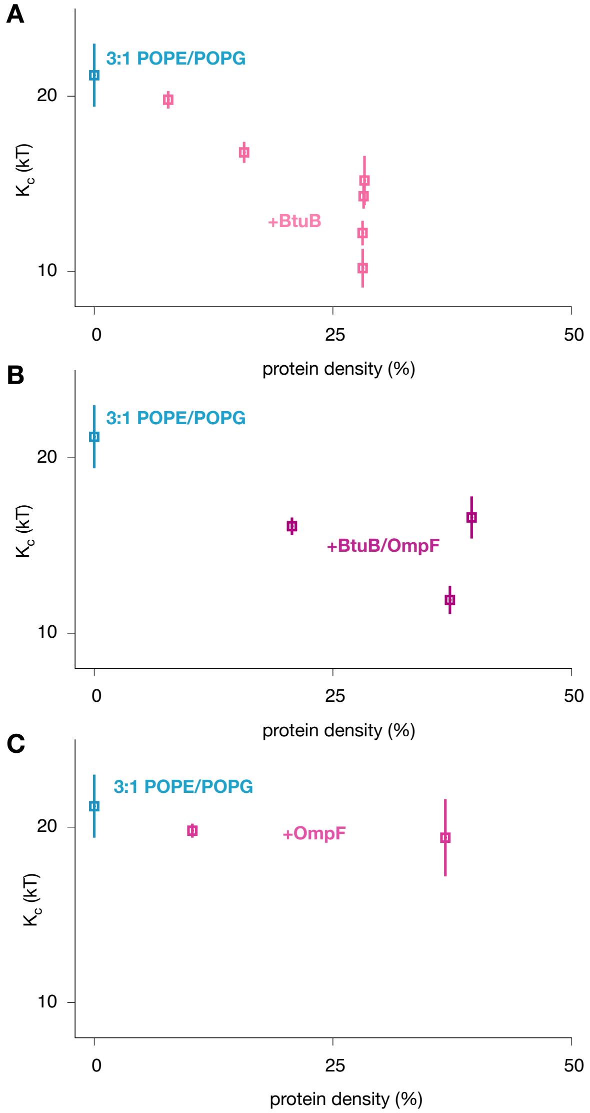

Figure S13: The bending rigidity of the membrane either is unchanged or decreases as the amount of protein added increases. The bending rigidity decreases when either (*A*) BtuB or (*B*) equal amounts of BtuB and OmpF are added to a POPE/POPG (3:1) lipid bilayer. (*C*) If only OmpF is added, there is no detectable change in the bending rigidity, K_c. A published series of coarse-grained simulations of smaller patches of POPE/POPG containing a range of densities of BtuB, OmpF and BtuB+OmpF[4] were analysed (Fig. S8-S11) and added to our dataset to improve the statistics. Details of these simulations can be found in Table S2.

References

1. J. B. Klauda, R. M. Venable, J. A. Freites, J. W. O'Connor, D. J. Tobias, C. Mondragon-Ramirez, I. Vorobyov, A. D. MacKerell and R. W. Pastor, *J Phys Chem B*, 2010, **114**, 7830–43.
2. S. J. Marrink, H. J. Risselada, S. Yefimov, D. P. Tieleman and A. H. de Vries, *J Phys Chem B*, 2007, **111**, 7812–24.
3. X. Periole and S.-J. Marrink, in *Methods in Molecular Biology*, ed. L. Monticelli and E. Salonen, Humana Press, Totowa, NJ, 2013, vol. 924, ch. 20, pp. 533–565.
4. P. Rassam, N. A. Copeland, O. Birkholz, C. Tóth, M. Chavent, A. L. Duncan, S. J. Cross, N. G. Housden, R. Kaminska, U. Seger, D. M. Quinn, T. J. Garrod, M. S. P. Sansom, J. Piehler, C. G. Baumann and C. Kleanthous, *Nature*, 2015, **523**, 333–336.
5. P. J. Bond and M. S. P. Sansom, *J Am Chem Soc*, 2006, **128**, 2697–704.
6. S. J. Marrink, A. H. de Vries and A. E. Mark, *J Phys Chem B*, 2004, **108**, 750–760.
7. D. V. Palanivelu, D. E. Kozono, A. Engel, K. Suda, A. Lustig, P. Agre and T. Schirmer, *J Mol Biol*, 2006, **355**, 605–611.
8. S. B. Hansen, X. Tao and R. MacKinnon, *Nature*, 2011, **477**, 495–8.
9. S. W. Cowan, R. M. Garavito, J. N. Jansonius, J. A. Jenkins, R. Karlsson, N. König, E. F. Pai, R. A. Pauptit, P. J. Rizkallah and J. P. Rosenbusch, *Structure*, 1995, **3**, 1041–1050.
10. V. Cherezov, E. Yamashita, W. Liu, M. Zhalnina, W. A. Cramer and M. Caffrey, *J Mol Biol*, 2006, **364**, 716–34.
11. J. B. Larsen, M. B. Jensen, V. K. Bhatia, S. L. Pedersen, T. Bjørnholm, L. Iversen, M. Uline, I. Szleifer, K. J. Jensen, N. S. Hatzakis and D. Stamou, *Nat Chem Biol*, 2015, **11**, 192.
12. W. Yang, R. Bitetti-Putzer and M. Karplus, *J Chem Phys*, 2004, **120**, 2618–2628.
